# Presence of Viable, Clinically Relevant *Legionella* Bacteria in Environmental Water and Soil Sources of China

**DOI:** 10.1128/spectrum.01140-21

**Published:** 2022-04-19

**Authors:** Xiao-Yong Zhan, Jin-Lei Yang, Honghua Sun, Xuefu Zhou, Yi-Chao Qian, Ke Huang, Yang Leng, Bihui Huang, Yulong He

**Affiliations:** a The Seventh Affiliated Hospital, Sun Yat-sen University, Shenzhen, China; University of Minnesota

**Keywords:** *Legionella*, *L. pneumophila*, *L. longbeachae*, environmental sources, soil, water

## Abstract

The distribution of pathogenic *Legionella* in the environmental soil and water of China has not been documented yet. In this study, *Legionella* was detected in 129 of 575 water (22.43%) and 41 of 442 soil samples (9.28%) by culture. Twelve *Legionella* species were identified, of which 11 were disease-associated. Of the *Legionella*-positive samples, 109 of 129 (84.50%) water and 29 of 41 (70.73%) soil were positive for L. pneumophila, which accounted for about 75% of *Legionella* isolates in both water and soil, suggesting L. pneumophila was the most frequent species. Soil showed a higher diversity of *Legionella* spp. as compared with water (0.6279 versus 0.4493). In contrast, serogroup (sg) 1 was more prevalent among L. pneumophila isolates from water than from soil (26.66% versus 12.21%). Moreover, many disease-associated sequence types (STs) of L. pneumophila were found in China. Intragenic recombination was acting on L. pneumophila from both water and soil. Phylogeny, population structure, and molecular evolution analyses revealed a probable existence of L. pneumophila isolates with a special genetic background that is more adaptable to soil or water sources and a small proportion of genetic difference between water and soil isolates. The detection of viable, clinically relevant *Legionella* demonstrates soil as another source for harboring and dissemination of pathogenic *Legionella* bacteria in China. Future research should assess the implication in public health with the presence of *Legionella* in the soil and illustrate the genetic and pathogenicity difference of *Legionella* between water and soil, particularly the most prevalent L. pneumophila.

**IMPORTANCE** Pathogenic *Legionella* spp. is the causative agent of Legionnaires’ disease (LD), and L. pneumophila is the most common one. Most studies have focused on L. pneumophila from water and clinical samples. However, the soil is another important reservoir for this bacterium, and the distribution of *Legionella* spp. in water and soil sources has not been compared and documented in China yet. Discovering the distribution of *Legionella* spp. and L. pneumophila in the two environments may help a deep understanding of the pathogenesis and molecular evolution of the bacterium. Our research systematically uncovered the distributions of *Legionella* spp. in different regions and sources (e.g., water and soil) of China. Moreover, phylogeny, population structure, and molecular evolution study revealed the possible existence of L. pneumophila with a special genetic background that is more adaptable to soil or water sources, and genetic difference may exist.

## INTRODUCTION

Legionnaires’ disease (LD) is an often severe and fatal form of pneumonia caused by pathogenic *Legionella* species (*Legionella* spp.), which is a group of Gram-negative, facultative, intracellular bacteria (1, 2). These bacteria are ubiquitously found in both aqueous environments and moist soils worldwide (3, 4). Although Legionella pneumophila (L. pneumophila) is the most common causative agent of LD, over 25 non-pneumophila *Legionella* spp. can cause disease (5, 6). Free-living protozoa in the environment are the protecting shelter for L. pneumophila and many other *Legionella* spp. From natural environments, L. pneumophila colonizes artificial water environments such as air-conditioning and hot-water systems and then spreads via aerosols (7, 8). Once inhaled, L. pneumophila could be phagocytosed by alveolar macrophages and replicate within them. Aspiration of contaminated natural water is another route of transmission. Soil is another potential source for *Legionella* and is determined to be associated with LD caused by *L. longbeachae* (9–12). Many studies also showed that L. pneumophila could inhabit commercial potting, natural, and garden soils (3, 4, 13, 14), suggesting soil as another potential source for L. pneumophila infection (15). Because person-to-person transmission of LD is rarely reported (16), LD is a specific disease transmitted by the environment, and the pathogenic strains hidden in the ecological niches are responsible for the disease (17).

To identify the source of *Legionella* infection, a genotypic match between environmental and clinical isolates is required. Although some sequence types (STs) of L. pneumophila were identified to be associated with Chinese LD cases, such as ST36, ST59, etc., few studies tracked the environmental sources of infection (18). Guo et al. and Qin et al. illustrated the distribution of L. pneumophila in natural and man-made water sources (19, 20), while there was no survey of soil as a source for pathogenic *Legionella* bacteria in China.

L. pneumophila is a rapidly evolving species with high genetic plasticity (21, 22). Infection in human beings implies the evolutive dead-end (16). Thus, long-term coevolution with free-living protozoa hosts provides the primary evolutionary pressure for the bacterium (8). The protozoa hosts act as gene melting pots and drive the acquisition of host cell genes and interexchange of bacterial genes for L. pneumophila through horizontal gene transfer, which can be evidenced by phylogenetic analysis (23, 24). It is established that L. pneumophila isolates from natural water (e.g., pools, lakes, rivers) had a higher genetic diversity than those from artificial water (cooling towers, hot water systems) and clinical samples, which supports a notion that only a small group of L. pneumophila could infect humans (25, 26). It is also puzzling why LD cases caused by *L. longbeachae* are nearly all associated with soil sources, though it is ubiquitously found in both environmental water and soil (9, 11, 12). In contrast, LD caused by L. pneumophila holds a close relationship with water isolates (27). It is not surprising that the diversity in species and quantity of protozoa are different between water and soil, which may affect the evolution of *Legionella* inhabited. Given that combinatorial selection in amoebal hosts drives the evolution of the L. pneumophila (28), the genome composition and genetic difference, as well as the pathogenicity, between L. pneumophila from natural water and soil sources may present. However, little attention has been paid to this topic.

The present study aims to investigate water and soil environments as reservoirs of viable, clinically relevant *Legionella* bacteria in China. More than 1,000 samples were collected throughout the year (2019 to 2021) from water and soil sources in different areas of China. Our results evidenced distinct distribution patterns of *Legionella* spp., serogroups (sgs), and STs of L. pneumophila between the water and soil sources of China, highlighting the role of soil as a reservoir for pathogenic *Legionella* spp. and clinically relevant STs of L. pneumophila. Furthermore, our study revealed the possible existence of L. pneumophila with special genetic backgrounds to be more adaptable to soil or water sources, and genetic differences between water and soil isolates.

## RESULTS AND DISCUSSION

### *Legionella* spp. from water and soil sources of China.

**FIG 1:**
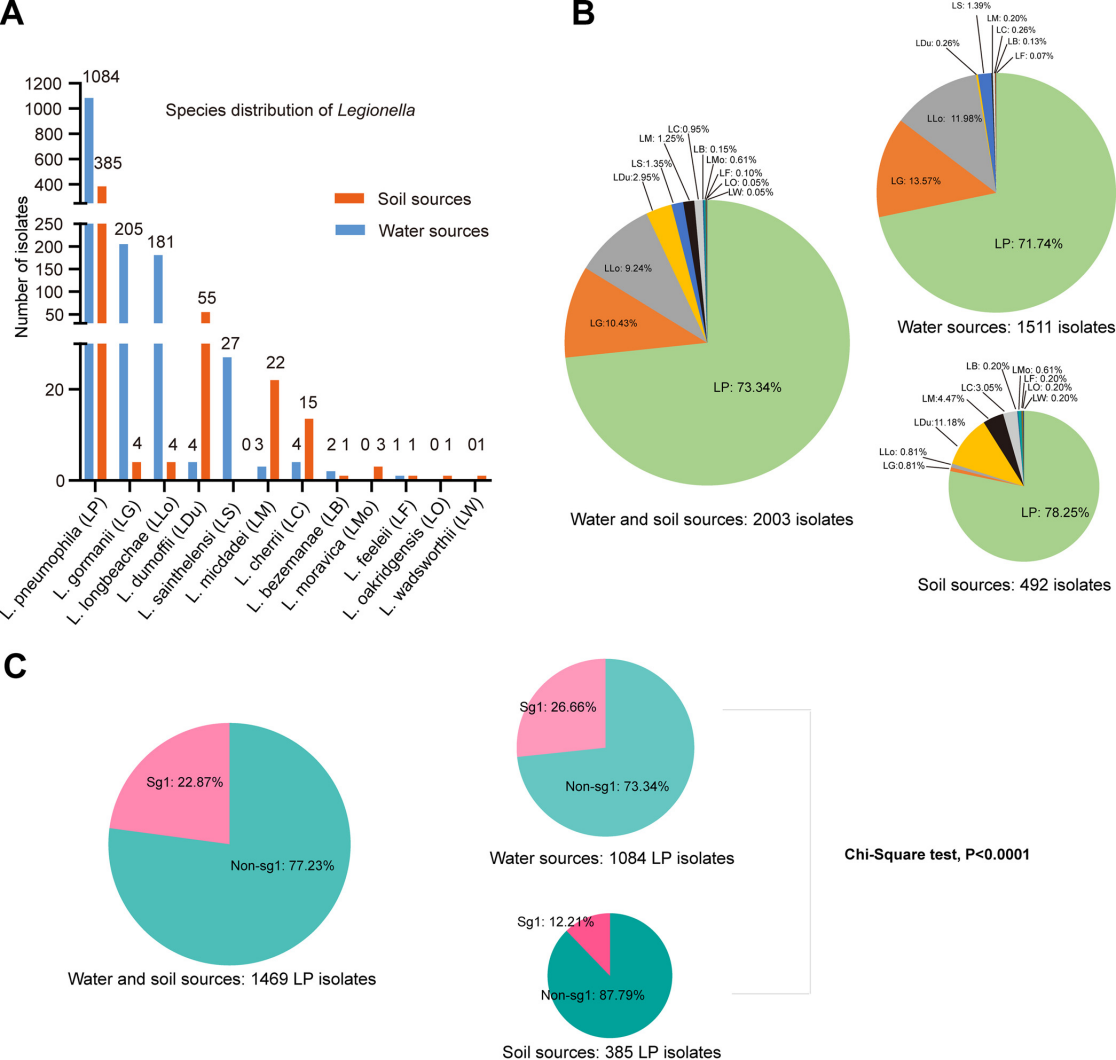
*Legionella* species distribution in the water and soil sources of China. (A) The numbers on the top of each bar indicate the quantity of *Legionella* from water or soil sources. (B) The composition of the *Legionella* spp. in different environments. (C) The composition of sg1 isolates among all *L pneumophila* in different environments.

A total of 575 water and 442 soil samples were collected, among which, 1,511 and 492 *Legionella* isolates were derived from 129 water and 41 soil samples, respectively. The details of the samples are shown in Table S1 in the supplemental material. A significantly higher positive rate for *Legionella* spp. was found in water (129/575, 22.43%) than in soil (41/442, 9.28%) by culture (*P* < 0.001, chi-square test) (Table 1). Garden soils showed a comparable positive rate (9.39%, 29/309) for *Legionella,* with a previous study showing a 12.43% (22/177) positive rate by using an amoebal coculture method (3, 4). The method has been proven successful for the isolation of *Legionella* in samples with a lot of background flora (e.g., soil samples) (29). This result indicated that our multiple quantity culture method worked effectively in soil *Legionella* isolation, for it could balance the quantity of background flora and *Legionella* bacteria. Fig. 1A shows the distribution of each *Legionella* spp. in environmental sources. Twelve species of *Legionella* were obtained in our sample collection, including L. pneumophila (accounting for 73.34% of all the isolates), *L. gormanii* (10.43%), *L. longbeachae* (9.24%), *L. dumoffii* (2.95%), *L. sainthelensi* (1.35%), *L. micdadei* (1.25%), *L. cherrii* (0.95%), *L. bozemanae* (0.15%), *L. moravica* (0.15%), *L. feeleii* (0.1%), *L. oakridgensis* (0.05%), and *L. wadsworthii* (0.05%), but the distribution of these *Legionella* spp. between water and soil sources was distinct (Fig. 1B). The Simpson diversities of *Legionella.* spp. in all samples and water or soil samples were 0.5586, 0.4493, and 0.6279, respectively, showing that soils had a higher diversity of *Legionella* spp. All the *Legionella* spp. we identified could cause infections, except *L. moravica* (30–39). Although there is no direct isolation of *L. cherrii* from human specimens, the metagenomic next-generation sequencing revealed it as an agent for severe community-acquired pneumonia in China (40). The distribution of *Legionella* spp. in the environments of other countries/regions and their association with clinical infections are summarized in Table 2. A seroepidemiology study of legionellosis in the mainland of China (1982–2014) showed that *L. micdadei*, *L. bozemanae*, *L. dumoffii*, and *L. longbeachae* account for 2.25%, 1.01%, 0.77%, and 0.24% of LD cases, respectively (41). Together with the distribution of non-L. pneumophila isolates in water and soil sources of China (Fig. 1 and Table 2), the results indicated that *Legionella* infection in China was not only caused by L. pneumophila but also by those non-L. pneumophila species that were stored in multiple environments, and highlight an urgent need for routine surveillance of *Legionella* in environmental sources. We also noticed that colony forming units (CFUs) of *Legionella* bacteria among water or soil samples from different sources or cities tend to be diverse (Fig. S1A–E). Large water areas (e.g., lakes, rivers, sea) had higher CFUs than small water areas (e.g., small streams, ponds, grassland puddles, and fountain) in general (Fig. S1A–B). Meanwhile, the higher the temperature the sample got, the higher the *Legionella* bacteria CFU of the sample was found, although the statistical difference did not reach significance (Fig. S1F–G).

**TABLE 1:** Numbers of L. pneumophila-positive, L. pneumophila sg1-positive, non-L. pneumophila-positive, and *L. longbeachae* -positive samples in the analyzed or *Legionella*-positive samples of each type of environmental source

Source type (no. of samples)	Mean temp (^°^C) (range)	No. of *Legionella*—positive samples (no. of L. pneumophila—positive samples) (no. of L. pneumophila sg1positive samples) (no. of non-L. pneumophila-positive samples) (no. of *L. longbeachae*-positive samples)	*Legionella* species-positive rates (L. pneumophila-positive rates,%) (L. pneumophila sg1-positive rates, %) (non-L. pneumophila-positive rates, %) (*L. longbeachae*-positive rates,) For analyzed samples	No. of L. pneumophila-positive samples (L. pneumophila-positive rates,%) For *Legionella*-positive samples No. of L. pneumophila sg1-positive samples (L. pneumophila sg1-positive rates,%) For *Legionella*-positive samples	No. of non-L. pneumophila-positive samples) (Non-L. pneumophila-positive rates,%) or *Legionella*-positive samples	No. of *L. longbeachae*-positive samples (non-*L. pneumophila-*positive-positive rates,%) For *Legionella*-positive samples
Water sources						
Small stream (198)	27.89 (23–34)	48 (44) (24) (10) (2)	24.24 (23.23) (12.12) (5.56) (1.01)	44 (91.66), 24 (50.00)	10 (20.83)	2 (4.17)
River (109)	28.14(14–34)	22 (14) (6) (14) (8)	20.18 (12.84) (5.50) (12.84) (7.34)	14 (63.64), 6 (26.09)	14 (63.64)	8 (36.36)
Lake (143)	27.83 (24–33)	34 (33) (18)(13) (7)	23.78 (23.08)(12.59) (9.09) (4.90)	33 (97.06), 18 (52.94)	13 (21.21)	7 (20.59)
Pond (91)	28.20 (16–33)	15 (10) (5) (6) (2)	16.48 (10.99) (5.49) (6.59) (2.20)	10 (66.67), 5(33.33)	6 (15.38)	2 (15.38)
Sea water (15)	30 (30)	5 (4) (3) (3) (2)	33.33 (26.67) (26.67) (20.00) (13.33)	4 (80), 3 (75)	3 (50)	2 (40)
Fountain (10)	27.33 (27–28)	3 (2) (2) (1) (0)	30 (20.00) (20.00) (10.00) (0)	2 (66.66), 2 (66.66)	1 (33.33)	0 (0)
Grassland Puddles (9)	31.5 (31–32)	2 (2) (2) (1) (0)	22.22 (22.2) (22.22) (11.11) (0)	2 (100), 2 (100)	1 (50)	0 (0)
Total water sources (575)	27.89 (14–34)	129 (109) (60) (49) (21)	22.43 (18.96) (10.43) (8.52) (3.65)	109 (84.50), 60 (46.51))	49 (37.98)	21 (16.28)
Soil sources						
Garden soil (309)	23.07 (13–31)	29 (22) (10) (13) (1)	9.39 (7.12) (3.24) (4.21) (0.32)	22 (75.86), 10 (34.48)	13 (44.83)	1 (3.45)
Potted soil (133)	22.53 (18–27)	12 (7) (1) (8) (2)	9.02 (4.51) (0.75) (6.02) (1.50)	7 (58.33), 1 (8.33)	8 (6.67)	2 (16.67)
Total soil sources (442)	23.07 (13–31)	41 (29) (11) (3)	9.28 (6.33) (2.49) (4.75) (0.68)	29 (70.73), 11 (26.83)	21 (51.22)	3 (7.32)

**TABLE 2:** The source and quantity of *Legionella* spp. with or without LD (based on literature reports) in this study^*a*^

Legionella spp.	Water sources							Soil sources		Other countries/regions with the same *Legionella* spp.	References
	Small stream	River	Lake	Pond	Puddle	Fountain	Seawater	Potted soil	Garden soil		
L. pneumophila	383	71	465	80	30	12	43	37	348	Worldwide (C, W, S)	(1, 59)
L. pneumophila sg1	137	22	80	17	16	2	15	2	45	Worldwide (C, W, S)	(1, 59)
L. gormanii	38	104	60	3					4	USA (C, S), China (W), Thailand (S)	(31, 46, 110)
L. longbeachae	3	105	53	3			17	3	1	Australia (C, S), Switzerland (C, W, S), Thailand (W), China (C), Japan (S), Denmark (C)	(9, 11, 14, 41, 45, 111–114)
L. dumoffii	3		1					12	43	Netherlands (C), USA (C), Canada (C, W), China (C), Denmark (C)	(33, 41, 47, 48, 114, 115)
L. sainthelensi	4	1	5	17						New Zealand (C), Canada (C), USA (W, C), Portugal (W), Thailand (S)	(49–54, 116)
L. micdadei		1	2					18	4	USA (W), Canada (C), UK (W, C), Switzerland (S), Denmark (C, W)	(14, 54, 55, 114, 117)
L. cherrii							4	15		USA (W), China (C), Demark (C, W)	(40, 118)
L. bozemanae		2							1	China (C), Denmark (C, W), Thailand (S)	(32, 41, 114, 116)
L. moravica									3	Denmark (W)	(114)
*L. feeleii*		1							1	Thailand (S), USA (C)	(116, 119, 120)
L. oakrigensis								1		USA(W), Denmark (W), France (C)	(114, 121, 122)
L. wadsworthii								1		USA (C)	(38)
Total (*n*)	Water (15,11)							Soil (492)			

a The numbers in each cell indicate numbers of isolates; blank cells indicate none. C, clinical-associated; W, isolated from water; S, isolated from soil.

### Distribution patterns of the most common pathogens L. pneumophila and *L. longbeachae* in China.

L. pneumophila and *L. longbeachae* account for most *Legionella* infections (1, 9). Of the 170 *Legionella* positive samples, 109 of 129 (84.50%) water and 29 of 41 (70.73%) soil samples were positive for L. pneumophila, respectively (Table 1), suggesting that L. pneumophila were more likely to store in water samples (*P* = 0.050, chi-square test). Similarly, L. pneumophila was the most common species in either water or soil sources, accounting for 71.74% (1084/1511) and 78.25% (385/492) of *Legionella* isolates in water and soil sources, respectively (Fig. 1B). Considering that L. pneumophila was found in all the nine types of samples (seven types of water samples and two types of soils) (Table 2), these results evidenced that L. pneumophila was the major and most widespread contamination of *Legionella* bacteria in both water and soil sources in China. However, we found bias distribution of sg1 L. pneumophila isolates between environmental water and soil sources (Table 1, Table 2, and Fig. 1C), although sg1 isolates could also be found in all nine types of samples. Among the 1469 L. pneumophila isolates, 336 were sg1, of which 289 were derived from water sources, and 47 were derived from soil sources (Table 2). They accounted for 26.66% (289/1084) and 12.21% (47/385) of L. pneumophila isolates from water and soil respectively (*P* < 0.0001, chi-square test). A previous study by Doleans et al. showed that sg1 accounted for 37.43% (776/2073) L. pneumophila isolates from man-made water sources including taps, showers, cooling towers, etc. in France (42). The geography and water source differences may lead to the different proportions of L. pneumophila sg1 in the environmental water. Isolation of sg1 from soil sources has been reported (13, 15, 43). However, the proportion of sg1 isolates in the soil has not been documented yet. The relatively lower proportion of sg1 isolates in the soil could be a partial explanation for why LD holds a close relationship with water, as sg1 was thought to be more virulent to humans than other sgs (44).

*L. longbeachae* was reported to account for about 30%–50% of LD cases in some countries such as Australia and New Zealand, and epidemiological investigations of these cases showed a close relationship with contaminated soil (9, 12, 30). We found *L. longbeachae* in 21 of 129 water samples (16.28%) derived from lakes, rivers, small streams, sea waters, and ponds, but not puddles and fountains (Table 1). Only 3 of 41 soil samples (both were potted soil, 7.32%) were positive for *L. longbeachae*. Moreover, 181 *L. longbeachae* isolates were from water samples, while only 4 were from soil samples (Fig. 1). These results suggested that water sources were more common shelters for *L. longbeachae* than the soil in China, which differed for countries such as Australia and New Zealand, where soils are the main reservoir for *L. longbeachae* (9). We also found that potted soils had a high positive rate for *L. longbeachae* than the garden soils (16.67% versus 3.45%, Table 1). In addition, the average number of *L. longbeachae* isolates per sample in the potted soils was about 6.35 times more than those in the garden soils (0.25 versus 0.034, Table 2). These results indicated commercial potted soils act as more crucial niches for *L. longbeachae* than the garden soils, which may be due to the special niche-fitness of *L.longbeachae* with plant materials (45).

### Distribution patterns of the *Legionella* spp. other than L. pneumophila and *L. longbeachae* in China.

*L. gormanii*, *L. longbeachae*, and *L. sainthelensi* were the second, third, and fourth common species in the water sources, respectively, whereas *L. dumoffii*, *L. micdadei*, and *L. cherrii* were the second, third, and fourth common species in the soil sources, respectively. Significantly different distribution patterns of the above six *Legionella* spp. between water and soil sources were documented (*P* < 0.01, Fisher’s exact test). *L. sainthelensi* was exclusively found in water samples, while *L. moravica*, *L. oakridgensis*, and *L. wadsworthii* were exclusively found in soil sources (Table 1, Table 2, and Fig. 1). *L. gormanii* was once isolated from a soil sample in the U.S. in 1978 and then found in a human bronchial brush specimen, which evidenced it as a human pathogen (31). It also caused mixed infection with L. pneumophila in humans (46). In this study, *L. gormanii* was the second most common pathogen of *Legionella* in the environments of China and distributed in many types of water or soil samples (Table 2), indicating the importance of surveillance of this bacterium. *L. dumoffii* was reported to be the fourth common pathogen of *Legionella* worldwide and was responsible for both intrapulmonary and extrapulmonary infections (47, 48). It was also the fourth common *Legionella* bacteria we identified. Given that it was frequently isolated and abundant in the soil samples (Table 2), particular attention should be paid to soil monitoring. *L. sainthelensi* caused many respiratory infections in New Zealand, Canada, and the U.S. (49–51), and was frequently distributed in water sources (52–54) but rarely distributed in soil sources (3), which was consistent with our current results, indicating water monitoring of this bacterium is urgently needed. *L. micdadei* was present in less than 1% of cases of community-acquired pneumonia (55) and was shown by our study to be more frequent and abundant in soil samples of China. *L. bozemanae* and *L. feeleii* were both found in water and soil samples, but their abundances were limited. *L. oakigensis*, and *L. wadsworthii* were only isolated in soil samples, further highlighting the importance of soil monitoring. Although *L. moravica* was isolated from a cooling tower, the absence of this bacteria in clinical samples suggested the association of this species with infection was not officially confirmed (56). There was no report of *L. moravica* contamination in soil sources previously, while we identified *L. moravica* in three garden soils of China.

### SBT sequence distribution and diversity in L. pneumophila isolates from water and soil sources.

Of the 1,469 L. pneumophila isolates, 471 relatively unrelated were selected for further analysis. These isolates comprised 263 water and 208 soil isolates. The SBT, a “gold standard” molecular genotyping method for L. pneumophila in the epidemiological investigation of LD was utilized to research the genetic association of these isolates (57, 58). Among the 471 isolates, 177 different SBT sequences were found with a Simpson’s index of diversity (IOD) value was 0.9860. Among the 177 STs, 128 (72.32%) were novel ones. The proportion of novel STs was comparable with the previous study in China (19). We found that water and soil L. pneumophila isolates had comparable ST diversity (0.9789 versus 0.9780) (Table S2). Among the 177 STs, 47 and 73 singletons were found in soil and water isolates, respectively. Fifteen SBT sequences were found in both water and soil isolates (Fig. S2). We only found that L. pneumophila isolates from soil sources had slightly lower diversities on *flaA* and *neuA/neuAh* loci (Table 3). These results indicated that coevolution of L. pneumophila in soil or water samples of China may be relatively consistent or frequently migration and gene exchange between the two types of isolates.

**TABLE 3:** Diversity of seven SBT loci of L. pneumophila isolates from water or soil sources

	Water sources (*n* = 263)			Soil sources (*n* = 208)		
Locus	No. of types	No. of isolates/type	Nei’s index^*a*^	No. of types	No. of isolates/type	Nei’s index^*a*^
*flaA*	19	13.84	0.87	13	16	**0.79**
*pilE*	23	11.43	0.87	17	12.24	0.86
*asd*	26	10.12	0.90	17	12.24	0.90
*mip*	32	8.22	0.91	23	9.04	0.92
*momps*	31	8.48	0.88	24	8.67	0.87
*proA*	25	10.52	0.90	17	12.24	0.90
*neuA/Ah*	32	8.22	0.92	21	9.90	**0.86**

a Nei's index of diversity as 1–Σpi^2^, where pi is the frequency of the SBT allele at the locus. The bold numbers indicate lower diversities on flaA and neuA/neuAh loci for the soil isolates.

### SBT sequences associated with LD.

Table 4 shows the sources and quantities of L. pneumophila isolates harboring STs associated with LD (based on literature reports). Many disease-associated STs were found in our isolate collection, including the ST1 (59), ST15 (60), ST461 (61), ST84, ST115, ST710, and ST48 (3, 62). Clinical associated STs in China such as ST36, ST59, and ST42 were not found in our environmental collection (18, 63). Previous studies demonstrated that soils were reservoirs of L. pneumophila ST47 strain, which caused many infections in the Netherlands and France (15, 64, 65). However, we did not find ST47 in either soils or waters. In contrast, disease-associated STs that have been previously found in soil isolates of the Netherlands, including the ST84, ST115, and ST710, were all found in our soil isolates collection (3). These STs (ST84, ST115, and ST710) were found regularly in garden soils and determined as soil-specific strains, and the ST84 isolates were the most common one (3), indicating soil as an ecological niche for L. pneumophila harboring these STs. ST84 was also previously isolated from composted material in the United Kingdom (66). In the present study, we identified six L. pneumophila isolates with ST84 sequences from three garden soils of Shenzhen city. ST115 was found in LD patients in the Netherlands (3). ST710 has been previously isolated from patients in Canada and Germany (4). Nine ST710 isolates were identified from a garden soil sample of Huangshi city. The relative abundance of disease-associated STs in soil sources further demonstrated that environmental soils serve as reservoirs and potential niches for pathogenic L. pneumophila bacteria in China, despite these STs having not been reported in clinical samples of China. Among the already identified STs, three ST22 isolates were found in two lake-water samples and a grassland puddle in Shenzhen city. ST22 was previously found in water samples from a bathtub and a bath sponge in Japan and caused sporadic cases of LD in Portugal (67, 68). ST384 isolates were found in Huizhou city. ST384 was shown to be associated with LD as it could be found in sputum specimens of patients with pneumonia (69). ST758, which was found in clinical samples in Italy in 2004 (70), was also found in a river water sample from Nanjing city. ST1712 was found in isolates both from water and soil including one sample from a grassland puddle of Guangzhou city, three samples from lakes, one sample from seawater, and a garden soil sample of Shenzhen city. ST1712 caused nosocomial neonatal legionellosis in Taiwan Province and was associated with infant formula (71). Combined with our findings, the widespread ST1712 isolates in different environmental sources might impose a high risk for infection. ST299 and ST18 were disease-associated STs found in France and Italy, respectively (72), and were also detected in lake and seawater isolates in Shenzhen city. ST367 and ST461 were reported as two closely related sg6 strain types isolated from patients and environmental water samples in the U.S. (73) and were detected in a garden soil sample in Shenzhen city and a lake water sample in Huizhou city, respectively. ST345, an ST that was associated with an outbreak of LD in Warstein, Germany in 2013, was also found in many man-made environmental water samples (74) but was less common in natural environmental samples, was found in a lake water sample from Dalian, a northeastern city in China. ST1119 was found in both cooling tower water and clinical samples in Catalonia, Spain (75). ST1119 was previously found in Macau (76) and was found in lake water in Huizhou city. ST739, previously found in clinical and water samples of Japan, and both natural and man-made water samples of China (19, 77), was found in a pond and a small stream in Shenzhen city, and a garden soil sample from Guangzhou city. ST74 was previously found in a hospital environmental swab in 2005 (78) and was associated with a cooling tower-associated outbreak of LD in Hong Kong in 2020 (79). ST763, one of the most prevalent environmental STs in the U.S. (69), was previously found in the environmental water of Guangzhou city, and patients in Japan (19). These two STs present in small streams and lake water samples in Shenzhen city, respectively. ST22 was previously found in soils, waters, and clinical samples (80, 81). We detected ST22 in a grassland puddle and lake water samples in Shenzhen city. ST506, previously found in a clinical sample in Japan (69), was found in a grassland puddle in Shenzhen city. ST1324, which corresponded to an sg8 clinical isolate in Canada, and water isolates in Canada, Italy, and Japan (82–84), was found in many sources including small streams, seawater, and garden soils in different areas of China. The presence of disease-associated STs in seawater confirmed it as a source of *Legionella* infection (85). A previous study also reported ST1324 in the environment in Greece and Kuwait (86). The abundance of isolates harboring ST1324 in many types of sources indicated wide adaptability. ST1439, corresponding to an sg10 isolate that caused LD in China (87), was found in the Yangzi River in Huangshi City in our study. Taken together, the wide spread of disease-associated STs in the water and soil suggested a high risk of LD in China, and routine surveillance of the two environments was necessary.

**TABLE 4:** The source and quantity of L. pneumophila isolates with STs associated with LD (based on literature reports) in this study^*a*^

STs	Water sources							Soil sources		Other countries/regions with the same SBT sequences	References
	Small stream	River	Lake	Pond	Puddle	Fountain	Sea water	Potted soil	Garden soil		
1	3									Worldwide (C, W)	(59)
15									1	Norway (C)	(60)
18			1				1			Italy (C), Norway (C, W)	(72)
22			2					2		Portugal (C, W), South Korea (W), Japan (S)	(67, 80, 81)
45	1	2	1				1		4	Canada (C), China (W), South Korea (W)	(19, 20, 123)
48	4	2	4				2		7	Belgium (C), UK (C, W)	(62, 124)
74	4									Hong Kong (C, W), France (W)	(78,79)
84									6	Netherlands (S)	(3)
115			1						4	Netherlands (S)	(125)
242		1								USA(C), China (W)	(125)
260		2	4	1				1	6	USA (C)	(126)
269								1		Italy (W), USA(C)	(126, 127)
299			1							Italy (C),	(72)
345		1								Netherlands (w), Germany (C, w), France (C)	(74)
367			1							USA (C, W),	(73)
384			3							Japan (W, C)	(69)
461								1	3	USA (C, W),	(73)
506					1					Japan (C)	(69)
579			1							Belgium (C), Norway (W), France (W), Netherlands (S)	(124, 128)
710									9	Netherlands (C, S)	(3)
739	1			1					1	Japan (C, W), China (W)	(19, 77)
758		1								Italy (C)	(70)
763			2							USA (W), China (W), India (W), Japan (C)	(19, 129–131)
1032		2								Japan (C), Taiwan (C, W)	(71, 77)
1119			1							Spain (C, W), Macau (W)	(75, 76)
1324	5		1				2		10	Canada (W, C), Japan (W), Italy (W)	(82–85)
1439		1								Greece (W), China (C), Kuwait (E)	(86, 87)
1694			4		1		6		2	New Zealand (C, W)	(132)
1712			4		1		1		5	Taiwan (C, W), Gabon (W)	(71, 133)
Total isolates	18	12	31	2	3	0	11	5	58	N/A	N/A
Total	77							63		N/A	N/A

a The numbers in each cell indicate numbers of isolates; blank cells indicate none. C, clinical isolates from patients; E, environmental isolates (exact sources, such as pools, lakes or rivers, not defined); W, isolates from water samples; S, isolates from soil samples; N/A, not available.

### Phylogenetic of L. pneumophila isolates based on SBT sequences.

**FIG 2:**
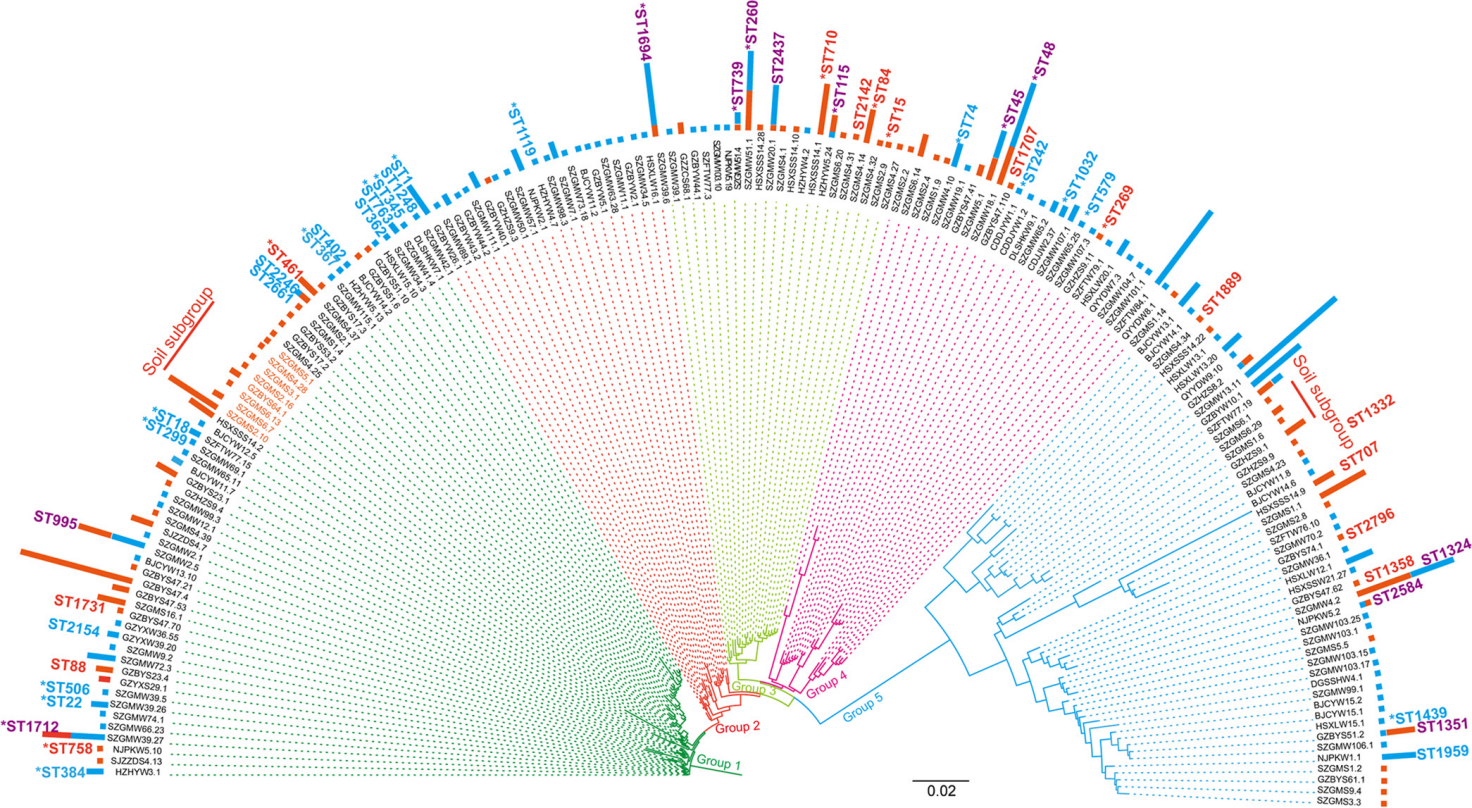
Phylogeny of 177 SBT sequences. The sequences’ names are shown as representative isolates’ name, and those that have definitive STs are shown in blue (isolates within the particular ST were all from water sources), orange (isolates within the particular ST were all from soil sources), and violet (isolates within the particular ST were from both water and soil sources). The length of the bar indicates the number of isolates with the same sequences. Branches that cluster into a clade are shown as a group and marked with the same color. Two phylogenetic closed soil subgroups are shown. Asterisks indicate that the STs are disease-associated.

**FIG 3:**
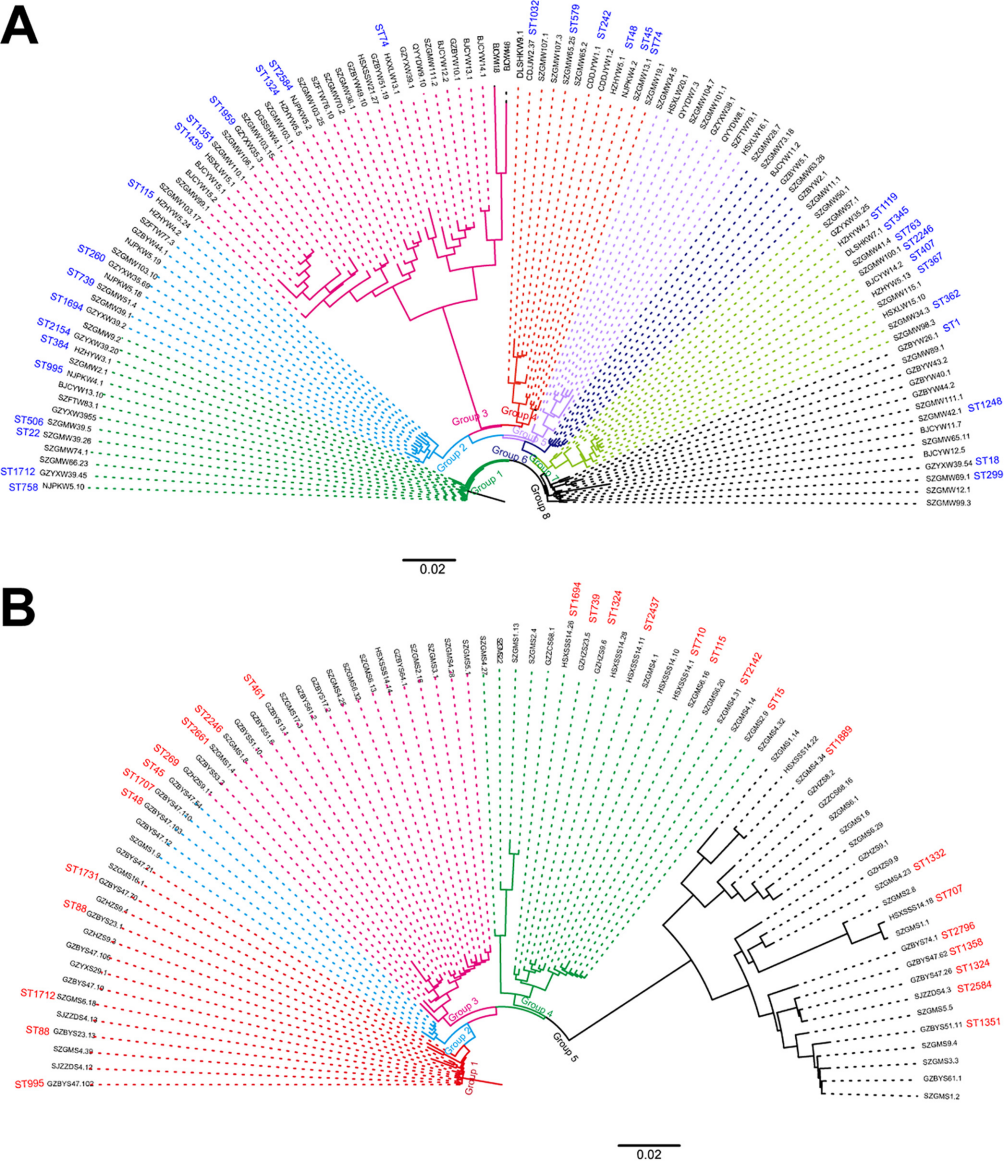
Phylogeny of SBT sequences from water isolates or soil isolates. (A) Phylogeny of SBT sequences derived from water isolates. (B) Phylogeny of SBT sequences derived from soil isolates. The sequences’ names are shown as representative isolates’ names, and those that have definitive STs are shown. Branches that cluster into a clade are shown as a group and marked with the same color.

The SBT sequences were shown as their representative isolate names. Some corresponding ST names for the sequences were marked, while some could not be obtained due to the accident of the webserver of the SBT database. We defined those ST names as C1, C2, C3, etc. (Table S3). As shown in Fig. 2, the Maximum Likelihood (ML) tree of the concatenated 177 SBT sequences showed 5 major groups. Group 1 constituted 58 SBT sequences containing isolates from both water and soil sources. Similar results were observed in groups 3, 4, and 5. These results indicated that some isolates from water and soil might have a similar phylogenetic history. However, we could determine some SBT sequences in these clades as soil subgroups (Fig. 2). Group 2 was relatively special, in that most (35/36, 97.22%) were water isolates, implying different phylogenetic patterns between some soil and water isolates. Eight main clades were classified in water isolates, while only five could be found in soil isolates (Fig. 3A and B), indicating that water isolates had a more complex phylogenetic history than soil ones.

### Population structure of water and soil isolates.

**FIG 4:**
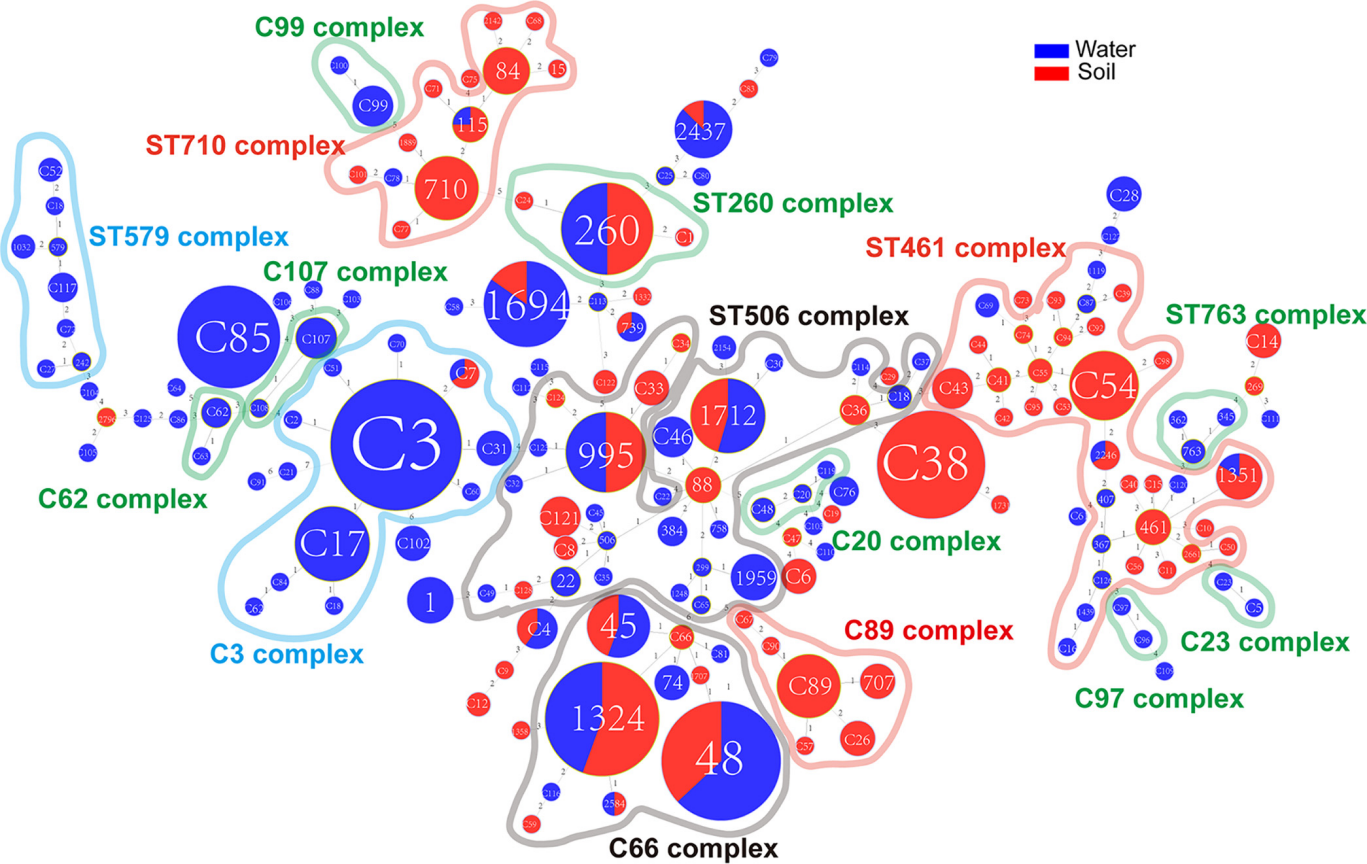
Minimum spanning tree of 471 environmental L. pneumophila isolates from China water and soil sources. STs are shown as circles. The size of each circle indicates the number of isolates from different types of sources within this particular ST. The shading rings simply link STs or sets of STs within an ST complex. Fifteen clonal groups were identified and named. Light red shading indicates isolates were mostly from soil sources (soil complexes), light blue shading indicates isolates were mostly from water sources (water complexes), gray shading indicates isolates were from both water and soil sources (mixed complexes), and light green shading indicates small water complexes.

**FIG 5:**
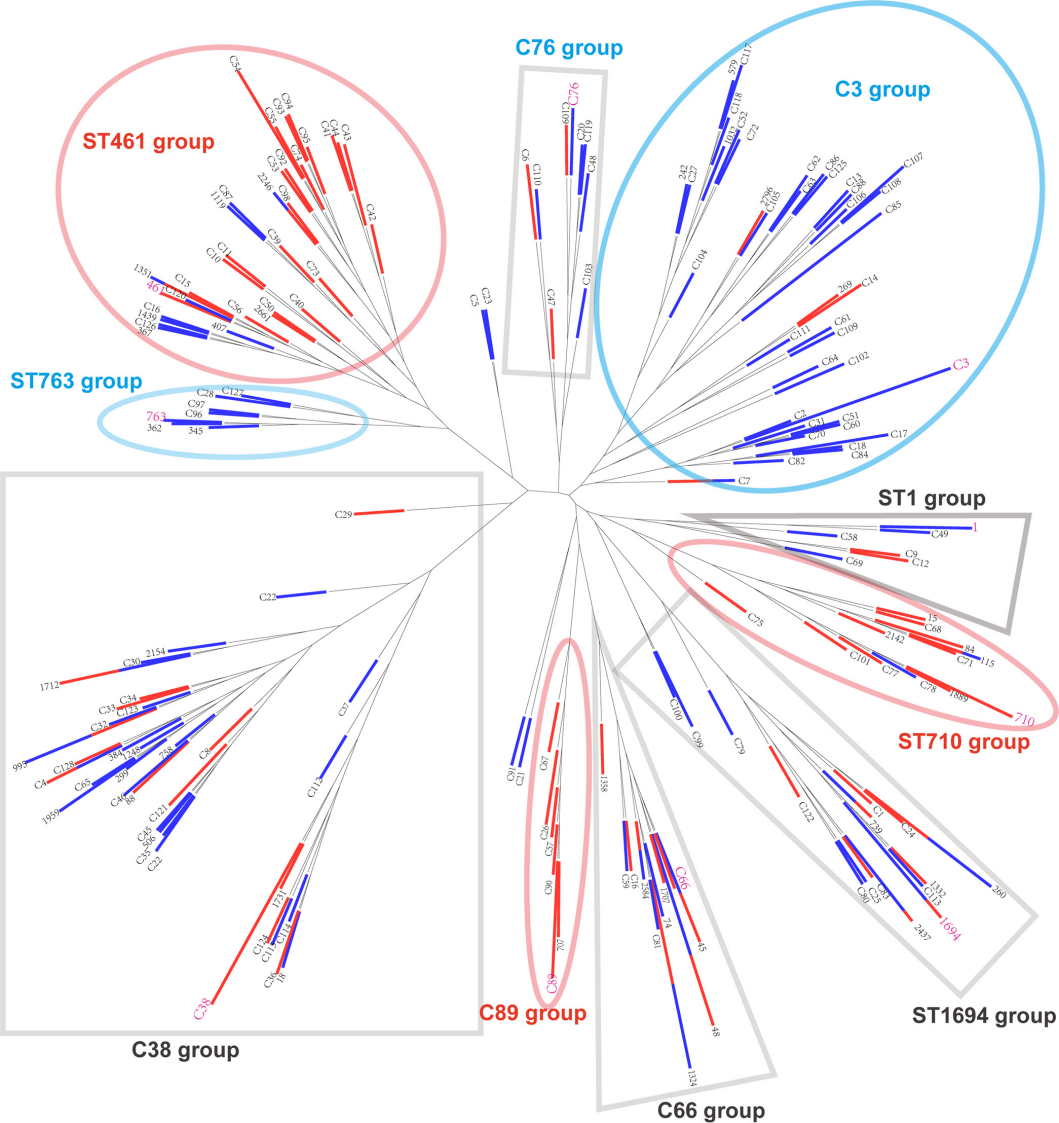
Neighbor-jointing tree of 471 environmental L. pneumophila isolates from China water and soil sources based on the profiles of SBT loci. STs are shown as sticks. The length of each stick indicates the number of isolates from different types of sources within this ST. The shading simply links STs or sets of STs within a group. Ten main groups were identified and named with representative isolates’ names (magenta). Red shading indicates that isolates were mainly from soil sources, while blue shading indicates that isolates mainly from water. Gray shading indicates isolates were from both water and soil sources.

**FIG 6:**
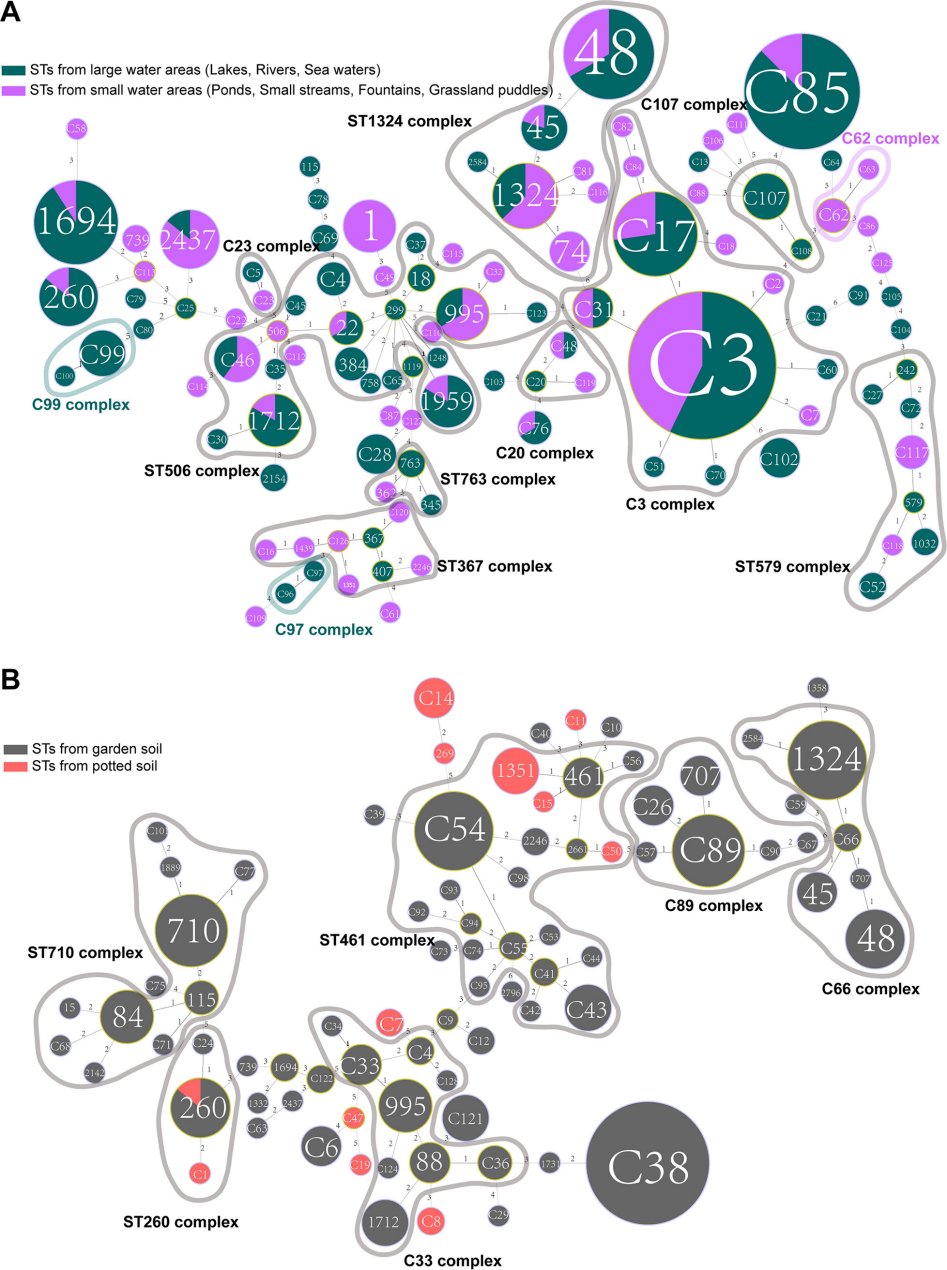
Minimum spanning tree of (A) 263 L. pneumophila water isolates, or (B) 208 soil isolates. STs are shown as circles. The size of each circle indicates the number of isolates from different types of sources within this ST. The shading rings simply link STs or sets of STs within an ST complex. Twelve clonal groups were identified and named in water isolates, five clonal groups were identified and named in soil isolates.

We utilized minimum spanning trees (MSTs) to illustrate the distribution of STs. As shown in Fig. 4, 177 STs were classified into 15 clone groups (complexes), whereas 40 STs, which differed from every other ST in three or more loci, were identified as singletons. We defined that a typical clonal complex should contain more than five STs. A complex was designated as either soil or water if the proportion of soil or water isolates harboring particular STs in the complex was greater than 75%. Finally, two typical water complexes (C3 and ST579) and three soil complexes (ST461, ST710, and C89) could be identified. Also, two mixed complexes (ST506 and C66) were found. These results suggested that some genetically associated STs tend to distribute in a particular environment, implying that L. pneumophila isolates with a special genetic background may be more adaptable to soil or water sources. An obvious example was the ST710 complex, which is made up of ST710, ST84, ST115, and some other novel STs in this study. The three STs mentioned here were found regularly in garden soils (3). ST461 in our study was another typical soil ST and a founder of many other STs from soil isolates, although it was found in water samples in a previous study (73). In contrast, the ST579 complex was typically waterborne, in which ST242 was previously found in water samples in China, and clinical samples in Japan and United Kingdom (19). ST1032 was found in clinical and water sources as well (71, 77). ST242 was previously identified as a singleton because it was not found in any clone complex of water isolates in China (19). It was defined as a member of the ST579 complex in our study, highlighting the importance of routine surveillance of L. pneumophila in environmental sources. The existence of mixed clone complexes might indicate the migration of L. pneumophila isolates between water and soil sources and genetic exchange, i.e., recombination among the isolates from the two sources. These results were also verified by the Neighbor-Jointing tree based on the allelic profiles of the 177 STs in which two typical water ST groups and three soil ST groups were identified (Fig. 5), although the members in the group were not always the same as the MST trees. We found 12 clonal complexes among STs derived from water sources and 6 from soil sources (Fig. 6, Table S4). These results further suggested distinct distributions of clonal complexes between water and soil isolates and tropisms of some complexes in either soil (e.g., C89, ST710) or water (e.g., C3, ST579) sources. ST1324, a founder ST of water isolates, was a member of C66 complex of all isolates. Similarly, C33, a founder ST of soil isolates, was a member of the ST506 complex of all STs. As C66 and ST506 complexes were all typical mixed ones, this result strengthens the notion that genetic exchange between the soil and water isolates might exist. However, we did not find specific complexes from potted soils (Fig. 6A), or large/small water areas (Fig. 6B), indicating frequent genetic exchange or migration of the L. pneumophila isolates within different water or soil sources.

### Molecular evolution of water and soil isolates.

**FIG 7:**
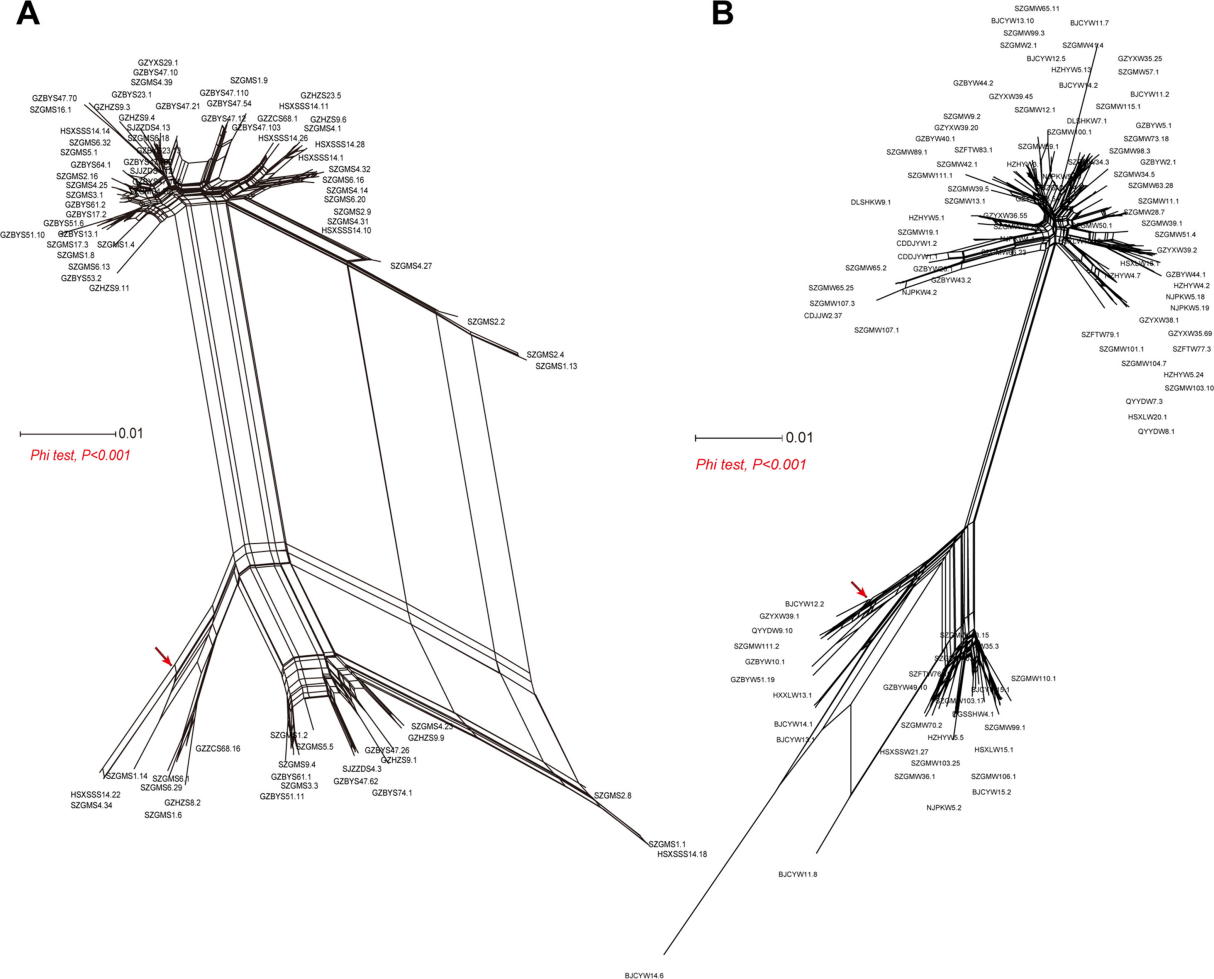
Reticulate network tree of SBT sequences from (A) soil isolates or (B) water isolates. All internal nodes represent hypothetical ancestral SBT sequences, and edges (examples are shown as red arrows) correspond to reticulate events such as recombination.

L. pneumophila was a bacterium with high genetic plasticity (21). Intragenic recombination played an important role in L. pneumophila evolution (23). As the natural hosts for L. pneumophila, the amoebae have distinct distribution between the water and soil sources (88, 89). Coevolved with natural hosts, the evolutionary forces acting on the isolates from the two sources might be discrepant (28). We therefore investigated the intragenic recombination in the two group isolates. A reticulate network tree obtained by the Neighbor-net algorithm of SplitsTree4 (90) using the concatenated SBT sequences from water or soil isolates made us visualize possible recombination. We found many edges that correspond to reticulate events such as recombination, apart from the internal nodes (Fig. 7). The implemented Phi test in SplitsTree4 did find significant evidence for recombination (both *P* < 0.001). By using RDP4, many recombination events were found not only on the water isolates but also on those from soil isolates. Twenty-two recombination events were found on 110 SBT sequences from water isolates, while 21 recombination events were found on 82 SBT sequences from soil isolates (Table 5). About one-fifth of major recombinant parents were not found in both group isolates, indicating that some of the isolates were not collected from the two sources or some isolates were not recovered during culture. Previous studies reported frequent recombination events at the single gene level or genome level of L. pneumophila from environmental water or clinical samples (22, 23, 91, 92). We reported here that L. pneumophila from soil sources was not a purely clonal origin but one undergoing significant recombination, similarly to those from clinical or water sources.

**TABLE 5:** Intragenic recombination among the SBT sequences from water or soil isolates by using six different methods implemented in the RDP software

Recombination events	Recombinant SBT sequence (STs)	Major parent (STs)^*c*^	Minor parent (STs)^*d*^	Detection methods implemented in RDP software^*b*^					
				RDP	GENECONV	Bootscan	Maxchi	Chimaera	SiSscan
STs from 263 water isolates									
1	GZBYW5.1^*a*^ (C18)	N/A	BJCYW14.6 (C21)	N^*e*^	Y^*f*^	Y	Y	Y	Y
2	GZYXW38.1 (C85)	HSXLW13.1 (C13)	BJCYW11.7 (C69)	Y	Y	Y	Y	Y	Y
3	QYYDW9.10 (C2)	N/A	SZGMW11.1 (C82)	Y	Y	Y	Y	Y	Y
4	GZYXW35.3 (C3)	N/A	SZGMW42.1 (1248)	Y	Y	Y	Y	Y	Y
5	QYYDW7.3 (C86)	BJCYW11.7 (C69)	GZBYW49.10 (C107)	Y	Y	Y	Y	Y	Y
6	GZYXW39.1 (C4)	SZGMW2.1 (C32)	BJCYW14.6 (C21)	Y	Y	Y	Y	Y	Y
7	BJCYW11.7 (C69)	BJCYW12.5 (C37)	N/A	Y	Y	Y	Y	Y	Y
8	GZBYW10.1 (C17)	SZGMW11.1 (C82)	BJCYW14.6 (C21)	N	Y	N	Y	Y	Y
9	BJCYW15.2 (C96)	SZGMW98.3 (C127)	BJCYW11.8 (C91)	Y	Y	Y	Y	Y	Y
10	BJCYW13.1 (C99)	SZFTW77.3 (C80)	BJCYW14.6 (C21)	Y	Y	N	Y	Y	Y
11	GZBYW51.19 (C102)	SZFTW79.1 (C64)	BJCYW14.6 (C21)	N	Y	Y	Y	Y	Y
12	BJCYW11.8 (C91)	BJCYW14.6 (C21)	GZBYW40.1 (C76)	Y	Y	N	Y	Y	Y
13	CDDJYW1.2 (C27)	SZGMW65.2 (C52)	SZGMW89.1 (C49)	Y	Y	N	Y	Y	Y
14	SZGMW65.2 (C52)	SZGMW100.1 (2246)	CDJJW2.37 (1032)	N	Y	N	Y	Y	Y
15	CDDJYW1.2 (C27)	SZGMW12.1 (C87)	N/A	Y	Y	N	Y	Y	Y
16	GZYXW35.25 (C28)	SZGMW98.3 (C127)	SZFTW77.3 (C80)	N	Y	N	Y	Y	Y
17	SZGMW12.1 (C87)	GZBYW26.1 (1)	BJCYW14.6 (C21)	N	N	N	Y	Y	Y
18	SZGMW103.25 (C115)	N/A	HZHYW4.2 (C78)	Y	Y	N	Y	N	Y
19	SZGMW99.1 (C109)	BJCYW15.2 (C96)	GZBYW49.10 (C107)	Y	Y	Y	Y	Y	Y
20	SZGMW42.1 (1248)	BJCYW12.5 (C37)	N/A	Y	N	N	Y	N	Y
21	SZGMW34.5 (C61)	SZGMW100.1 (2246)	GZBYW49.10 (C107)	N	N	N	Y	Y	Y
22	SZGMW107.3 (C118)	CDJJW2.37 (1032)	SZGMW100.1 (2246)	N	Y	Y	Y	Y	Y
STs from 208 soil isolates									
1	SZGMS1.13 (C26)	GZBYS51.1 (1351)	N/A	Y	Y	Y	Y	Y	Y
2	SZGMS2.2 (C67)	GZBYS47.2 (C29)	HSXSSS14.18 (707)	Y	Y	Y	Y	Y	Y
3	SZGMS5.5 (C124)	GZYXS29.1 (C8)	HSXSSS14.18 (707)	Y	Y	Y	Y	Y	Y
4	GZBYS51.11 (1351)	GZBYS51.10 (C11)	HSXSSS14.18 (707)	Y	Y	Y	Y	Y	Y
5	SJZZDS4.3 (2584)	GZBYS47.54 (45)	HSXSSS14.18 (707)	Y	Y	Y	Y	Y	Y
6	SZGMS4.27 (C75)	GZBYS53.2 (C50)	N/A	Y	Y	Y	Y	Y	Y
7	GZHZS9.1 (C14)	GZHZS9.1 (C14)	HSXSSS14.18 (707)	N	Y	N	Y	Y	Y
8	GZHZS9.9 (C1)	HSXSSS14.26 (1694)	SZGMS2.8 (C90)	Y	Y	Y	Y	Y	Y
9	SZGMS2.8 (C90)	N/A	GZBYS53.2 (C50)	Y	Y	Y	Y	Y	Y
10	HSXSSS14.22 (C101)	N/A	GZBYS47.70 (1731)	Y	Y	Y	Y	Y	Y
11	SZGMS1.14 (C98)	N/A	SZGMS2.9 (15)	Y	Y	Y	Y	Y	Y
12	SZGMS6.1 (C9)	GZBYS51.10 (C11)	HSXSSS14.18 (707)	N	Y	Y	Y	Y	Y
13	GZBYS47.70 (1731)	GZYXS29.1 (C8)	HSXSSS14.18 (707)	Y	Y	Y	Y	Y	N
14	GZBYS53.2 (C50)	SZGMS5.1 (C73)	HSXSSS14.18 (707)	Y	Y	N	Y	Y	N
15	SZGMS2.2 (C67)	SZGMS5.1 (C73)	HSXSSS14.18 (707)	N	Y	Y	Y	Y	Y
16	SZGMS16.1 (C121)	GZYXS29.1 (C8)	N/A	Y	N	N	Y	Y	Y
17	SZGMS5.1 (C73)	GZBYS51.10 (C11)	SZGMS2.9 (15)	N	Y	Y	Y	Y	Y
18	GZHZS9.11 (269)	N/A	SZGMS1.9 (C59)	Y	Y	N	Y	Y	Y
19	GZBYS51.10 (C11)	HSXSSS14.14 (C43)	N/A	N	Y	Y	Y	Y	Y
20	GZHZS9.1 (C14)	SZGMS2.9 (15)	SZGMS1.13 (C26)	Y	Y	N	N	N	Y
21	HSXSSS14.14 (C43)	GZHZS9.3 (C19)	GZBYS51.6 (C15)	N	N	N	Y	Y	Y

a The SBS sequence names are shown as their representative isolates’ names.

b Recombination events detected by more than two methods are shown.

c Major parent: parent SBT sequences contribute the larger fraction of the sequence.

d Minor parent: parent SBT sequences contribute the smaller fraction of the sequence.

e N indicates recombination events were not detected by the selected method.

f Y indicates recombination events were detected by the selected method.

Based on the coding region of the concatenated SBT sequences, significantly positive values of Fu and Li’s D*&F* but not the Tajima’s D were found in the soil isolates, suggesting a deficit of recent mutations in the SBT loci of soil isolates, and these mutations have occurred in the older part of the genealogy (Table 6). Further study demonstrated that the *pilE* and *asd* loci hold for recent mutations in the soil isolates, while the *mip* locus experienced farthest mutations that generate an excess of singletons in the water isolates (Table S5) (93). We also found extremely low nonsynonymous mutations (*dN*) but comparable synonymous mutations (*dS*) in the *proA* locus of the soil isolates (Table S5). Taken together, these results indicated that evolutionary patterns between the water and soil isolates might be partially different which may cause genetic differentiation.

**TABLE 6:** Summary of genetic diversity parameters for L. pneumophila isolates from water and soil sources based on the concatenated SBT sequences^*a*^

Parameters	Water isolates	Soil isolates
Sequences, *n*	263	208
Haplotypes, h	110	82
Haplotype diversity, Hd	0.9789	0.9780
Nucleotide diversity, π	0.04015	0.04000
SD	0.00131	0.00216
Polymorphic sites, S	459	377
Theta per site (from S)	0.03002	0.02564
SD	0.00628	0.00558
Avg no. of nucleotide differences, k	99.853	99.469
Total no. of mutations, Eta	546	444
*dN*	0.02171	0.01981
*dS*	0.1161	0.1244
*dN/dS*	0.1870	0.1592
Tajima’s D	0.39114	1.03646
Fu and Li’s D	0.34816	1.91565**
Fu and Li’s F	0.43781	1.75505*

a To fit the codon frame for calculating the *dN* and *dS*, 11 bp of nucleotide was removed from the SBT sequences including locus *flaA*, 2 bp; *asd*, 2 bp; *mip*, 3 bp; *and momps*, 4 bp. The bold numbers indicate significant neutrality indices of soil isolates. *, *P* < 0.05; **, *P* < 0.02.

A hierarchical analysis of molecular variance (AMOVA) for the 471 L. pneumophila isolates based on concatenated SBT sequences showed that the largest proportion of the genetic variation was found within populations, accounting for 96.62% of the total variation (Table 7). The proportion of the total genetic variation explained by differences between water and soil isolates was relatively small and accounted for 3.38% of the total variation, but it was significant (Table 7). Significant values of fixation index among populations (FST) were found, supporting that genetic differentiation exists between water and soil isolates. This could also explain the existence of water and soil-specific clades in the phylogenetic tree or clone complex found in our study (Fig. 2, 4, and 5). To eliminate the possible influence of geography differences on genetic differentiation, we took isolates from Guangzhou and Shenzhen cities into further analysis. Isolates from the two cities were assigned into two groups (Guangzhou and Shenzhen), and isolates in each group were assigned into two populations (water and soil). The fixation index among groups (FCT) was −0.2791, and the variation components did not vary significantly among the groups (*P* = 0.66178), implying no significant difference in genetic differentiation of isolates from these two cities (Table S6). In contrast, the fixation index among populations (FSC) was 0.08218, and genetic differentiation varied significantly among populations (*P* < 0.0001) (Table S6), further proving the existence of differentiation between isolates from soil and water.

**TABLE 7:** Summary of AMOVA results^*a*^

Source of variation	Sum of squares	Variance components	Percentage variation	Fixation index	Value	*P* value
Among populations	461.082	1.768	3.38	FST	0.03384 (Vc)	<0.0001
Within populations	23667.28	50.465	96.62	N/A^*b*^	N/A	N/A
Total	241287.97	52.232	100	N/A	N/A	N/A

a AMOVA testing included a single group consisting of all isolates, and these isolates were split into two populations (water and soil isolates).

b N/A, not available.

## MATERIALS AND METHODS

### Ethics statement.

There were no specific permissions required for the collection of water or soil samples from lakes, rivers, and ponds, or gardens, etc., because they were public open areas for citizens. Our study did not involve endangered or protected species.

### Sampling and processing of water and soil samples.

From April 2019 to January 2021, 575 water samples and 442 soil samples were collected from 16 cities in 11 provinces of China. The water samples were from rivers, lakes, ponds, small streams, fountains, grassland puddles, and sea waters. The soil samples were from potted soils (determined as soils directly collected from potted plants) and garden soils (determined as soils in the garden, including natural soil and compost). Isolation of *Legionella*-like bacteria from water samples was performed by a modified procedure from our previous report (94). Briefly, 250 mL water was concentrated by filtration through a suction filter (Model R300, Sciencetool., USA) with 0.45 μm membrane (Shanghai Xinya., China). Deposition on the filter membranes was scraped and washed to release the microorganisms and transferred into a sterile tube with ∼1.5 mL ddH_2_O. For soil, 10 g of moist samples were collected into a 50 mL sterilized centrifuge tube and transferred to the laboratory within 24 h, then they were suspended with 40 mL ddH_2_O. After a 2-min violent shock and 48 to 72 h standing, the supernatant was transferred into the suction (Model R300, Sciencetool., USA) with 0.45 μm membrane to concentrate the sample, and the microorganisms were released into ∼1.5 mL ddH2O. And finally, 250 mL water and 10 g soil samples were concentrated and suspended in 200 μL sterile water for culture after acid treatment (0.2 M HCl–KCl, pH 2.2 for 10 min) and heat treatment (50°C for 30 min).

### Isolation of *Legionella*-like bacteria.

We used a multiple quantity culture method to isolate *Legionella*-like bacteria from both water and soil samples. For the 200 μL processed samples, 32 and 128 μL (1:4) were plated onto two BCYEɑ-GVPC plates, respectively. The treatment was to eliminate non-*Legionella* organisms. Plates were incubated at 37°C with 5% CO_2_ (vol/vol) atmosphere for 3 to 7 days. *Legionella*-like morphology colonies were transferred to BCYEɑ-agar (with l-cysteine) and BCYEɑ-cys agar (without l-cysteine) for further determination. *Legionella* isolates were then identified to the species level by amplification and sequencing partial *16S rRNA* gene using universal primers 27F/1492R and amplification of *mip* gene using primers mip_F: 5′-GGG(AG)ATT(ACG)TTTATGAAGATGA(AG)A(CT)TGG-3′ and mip_R: 5′-TC(AG)TT(ATCG)GG(ATG)CC(ATG)AT(ATCG)GG(ATCG)CC(ATG)CC and sequencing *mip* using mip_fs: 5′-TTTATGAAGATGA(AG)A(CT)TGGTC(AG)CTGC-3′ (the underlining indicates degenerate bases), which were reported elsewhere (95–97). The *wzt/lpg0773* gene located in the cluster for lipopolysaccharide (LPS) biosynthesis was shown to be a part of sg1 specific gene regions that presented only in sg1 isolates based on a DNA-array analysis of 249 strains belonging to 15 different L. pneumophila sgs; thus, it was used as a marker to genetically identify sg1 as reported (98–100). Therefore, we designed a PCR method targeting the sg1-specific *wzt* gene for sg1 identification. The primers were: sg1-wzt_F: 5′-GCCACTGCCTTCATCCATT-3′ and sg1-wzt_R: 5′-CGCAAAGCCCAGAAATGAT-3′ based on our *in-silico* analysis of nine reference sg1 strains (Fig. S3). The flow chart of sampling and isolation of *Legionella* from water and soil sources is shown in Fig. S4.

### Obtaining SBT sequences.

The SBT sequences of each L. pneumophila isolate were determined by using the standard protocol from The European Study Group for *Legionella* Infections (ESGLI) with seven gene fragments (*flaA*, *pilE*, *asd*, *mip*, *mompS*, *proA*, and *neuA/neuAh*). The PCR products of the seven loci were sent to Guangzhou IGE Inc. for purification and sequencing. The quality of DNA sequencing was checked by SnapGene-viewer (https://www.snapgene.com/). The novel alleles of the SBT loci and sequences were determined by PHE (Public Health England) staff because of the accident or misconfiguration of the SBT web server (http://bioinformatics.phe.org.uk/legionella/legionella_sbt/php/sbt_homepage.php).

### Sequence analysis.

Sequence alignments were performed using the MUSCLE algorithm implemented in MEGA-X (101). Phylogenetic analysis was conducted by a Fasttree2 package based on ML method (102). The tree was drawn to scale, with branch lengths in the same units as those of the evolutionary distances used to infer the phylogenetic tree. The tree nodes were evaluated by bootstrapping with 1,000 replicates.

### Population structure analysis.

Hunter and Gaston’s modification of Simpson’s index of diversity (IOD) of STs was calculated by using the allele profiles of the isolates (103). The proportion of each ST was compared between water and soil isolates using Chi-square or Fisher’s exact tests. The Global Optimal eBURST (goeBURST) algorithm (104), as implemented in PHYLOViZ (105), was used to create an MST tree. In MST, the founder ST was defined as the one with the highest number of single-locus variants and was designated as a clone complex name. Clusters of related STs that descend from a common ancestor are defined as clone complex; single genotypes that do not correspond to any clone groups are defined as singletons. STs are represented by circles; the size of a circle shows the number of isolates of this type (Fig. 4 and 6). The number of variants between the two types is indicated in the connecting lines. Neighbor-Jointing tree of the obtained STs was created by using the Studier-Kepper Criterion (Hamming distance) algorithm implemented in PHYLOViZ (105).

### Molecular evolution analysis.

The SBT sequences were screened using RDP4 to detect intragenic recombination (106). Six methods (RDP, GENECONV, BootScan, MaxChi, Chimaera, and SiScan) implemented in the program RDP4 were utilized (106). Potential recombination event (PRE) was considered as that identified by at least three methods. Common settings for all methods were to consider sequences as linear, and statistical significance was set at the *P* < 0.05 level. The neighbor-net analysis was performed and converted to a split graph using the drawing algorithms implemented in SplitsTree4 software (version 4.14.4) (90). A reticulate network tree was prepared to show the relationships among different STs and to visualize possible recombination events. The *Phi* test implemented in Splitstree4 was used to statistically define recombination (90).

Genetic diversity analysis was performed using DnaSP v6 (107). Several statistical analysis-based methods implemented in the DnaSP were conducted to identify departures from the neutral model of evolution, including Tajima’s D, Fu, and Li’s D* &F* tests (108, 109). Hierarchical analysis of molecular variance (AMOVA) for SBT sequences from the two environments was performed by using Arlequin Ver3.5.2. This analysis provides estimates of variance components and F-statistics analogs speculating on the correlation of haplotype diversity at different levels of the hierarchical subdivision. We defined the hierarchical subdivision of these isolates at two levels. At the upper level, the two groups considered were water and soil isolates. The second level corresponded to the different haplotypes that were found within the two groups considered in the previous level. The statistical significance of fixation indices was tested using a nonparametric permutation approach.

### Statical analysis.

The Mann–Whitney U test was applied to evaluate the differences in quality of obtained isolates between soil and water sources. Moreover, the chi-squares or Fisher’s Exact tests were used for comparing the proportion of categorical variables. The statistical analysis was conducted with SPSS 25.0 (IBM software). GraphPad Prism version 8.0 (San Diego, CA, USA) was used for graphing. Statistical significance was defined as *P* < 0.05.

### Data availability.

The SBT sequences from L. pneumophila environmental isolates determined in this study were deposited in the GenBank Nucleotide Sequence Database with accession numbers MZ063063 to MZ063533.
