# Supplementary material for: Presence of Viable, Clinically Relevant Legionella Bacteria in Environmental Water and Soil Sources of China
Source: Microbiol Spectr. 2022 Apr 19;10(3):e01140-21. doi: 10.1128/spectrum.01140-21 (PMC9241679; doi:10.1128/spectrum.01140-21)
Supplement: SUPPLEMENTAL FILE 1 — Supplemental material. Download spectrum.01140-21-s0001.pdf, PDF file, 2.6 MB [file spectrum.01140-21-s0001.pdf]

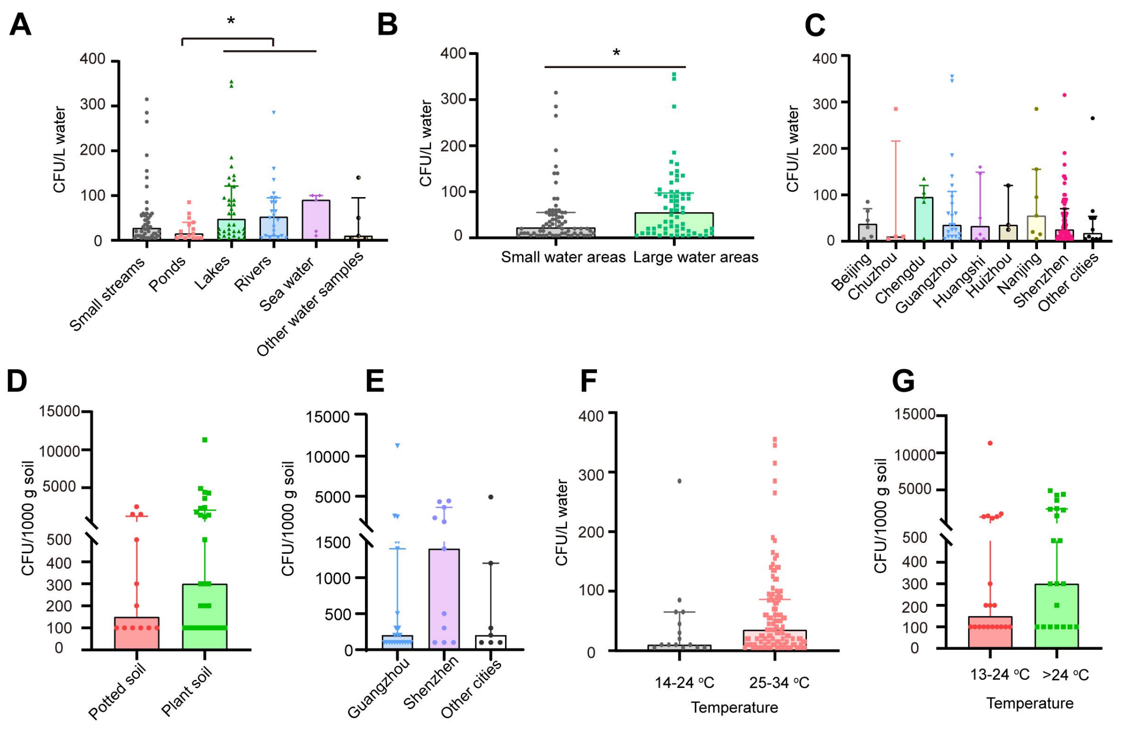

Fig. S1. CFU of *Legionella* bacteria in different environmental sources or cities, or isolated at different temperatures. A-B. CFU of *Legionella* bacteria in different water sources. **C**. CFU of *Legionella* bacteria in water sources of different cities. **D-E.** CFU of *Legionella* bacteria in different soil sources or soils from different cities. **F-G**. The higher temperature of the water and soil sources may indicate higher CFU of *Legionella* in the samples. The data are shown as scatter dot plots and median with 75% interquartile. * *P<0.05*.

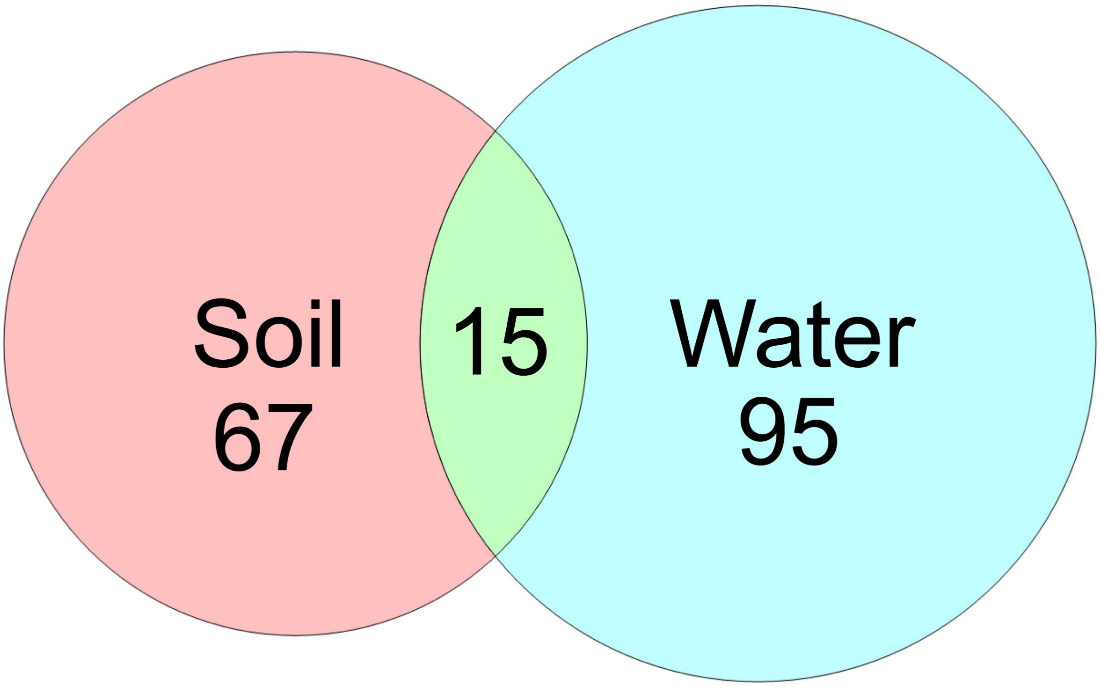

Fig. S2. Venn diagram shows the number of STs shared between isolates from different environmental sources of China.

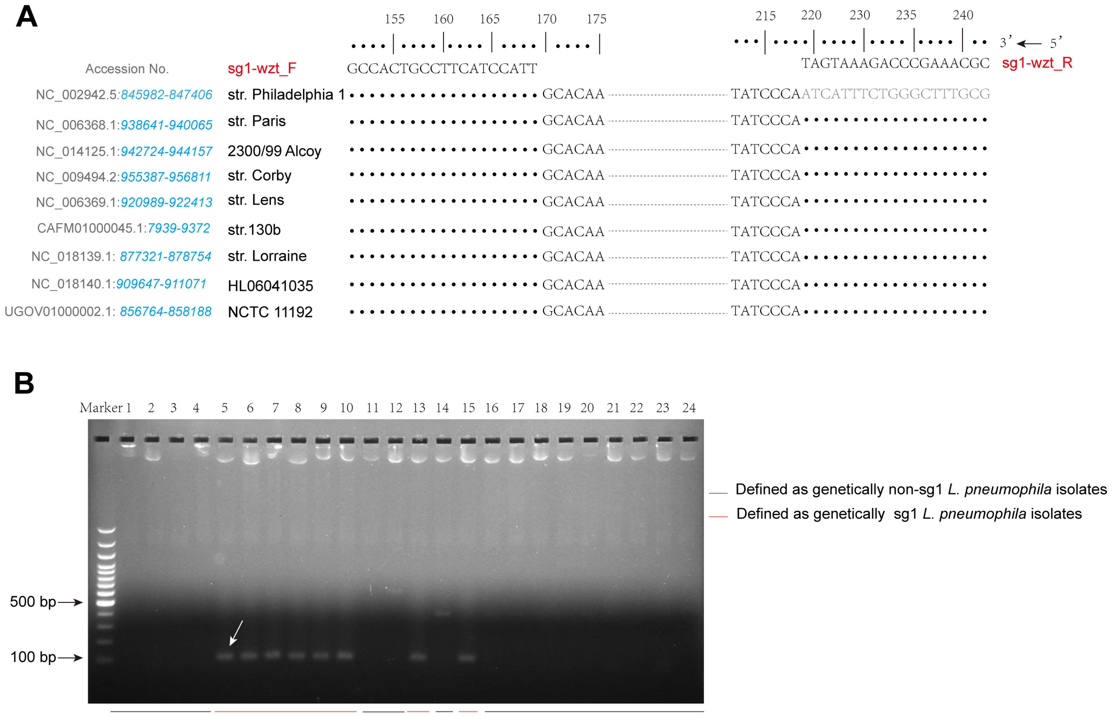

Fig. S3. Alignment of sg1 *wzt* primers with DNA sequences of *wzt* gene from *L. pneumophila* sg1 strains, and PCR for sg1 identification. A. Sg1-wzt primers and DNA sequences of sg1 specific *wzt* gene, Genbank accession number are shown and the blue italics indicate the sg1 specific *wzt* gene location in the selected sequences of the strains. **B**. Partial amplification of the sg1 specific *wzt* of *L. pneumophila* isolates using a pair of sg1-*wzt* primers. Electrophoresis in 2% agarose gels with ethidium bromide staining (0.5 μg/mL). DNA electrophoresis shows sg1 specific *wzt* target (~100 bp) for some isolates, and these isolates were genetically identified as sg1. The following *L. pneumophila* isolates were used (from lane 1 to lane 24): water isolates, SZGMW4.7, SZGMW4.8, SZGMW4.9, SZGMW4.10, <u>SZGMW5.1</u>, <u>SZGMW5.2</u>, <u>SZGMW5.3</u>, <u>SZGMW5.4</u>, <u>SZGMW5.5</u>, <u>SZGMW5.6</u>, SZGMW9.2, SZGMW11.1; soil isolates: <u>GZHZS9.4</u>, GZHZS9.5, <u>GZHZS9.6</u>, GZHZS9.7, GZHZS9.8, GZHZS9.9, GZHZS9.10, GZHZS9.11, GZHZS9.12, GZHZS9.13, GZHZS9.14, GZHZS9.15. The white arrow indicates sg1 specific *wzt* gene exists, and isolates with name marked in the underline were genetically identified as sg1.

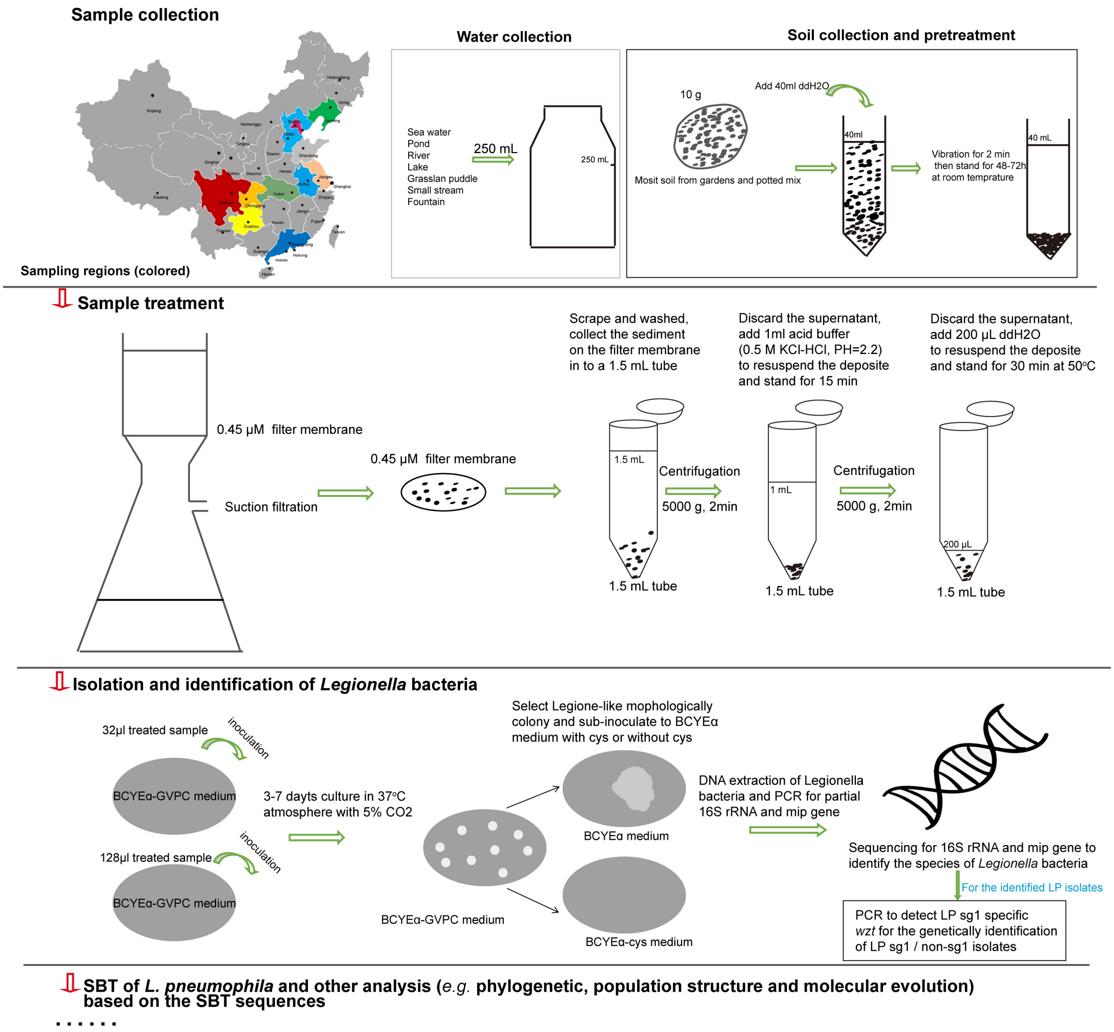

Fig. S4. Flowchart of this study. The flowchart describes the procedure of environmental sample collection, treatment, and isolation, and identification of *Legionella* bacteria.

Table S1. The details of collected samples, including the sources, sampling time and regions, Legionella species isolated, and quantity of isolates from the sample.

Serial no.	Sample no.	Sources of sample	City& City regione	Longitude and latitude (N, E)	Sampling time (yyyy-mm-dd)	**Legionella* species found in the sample	Tempratur e (centigrad	Quantity of isolates from the sample	Legionella Cfu of each sample (cfu/L water or cfu/kg soil)
Water	Water isolates								
1	SZGMW2	Small stream	Shenzhen, Guangming	22.7848, 113.9505	2019-04-26	LP	27	11	55
2	SZGMW4	Small stream	Shenzhen, Guangming	22.7844, 113.9510	2019-04-26	LP	27	9	45
3	SZGMW5	Small stream	Shenzhen, Guangming	22.7852, 113.9505	2019-04-26	LP$	27	6	30
4	SZGMW7	Small stream	Shenzhen, Guangming	22.7850, 113.9516	2019-04-26	LP$	27	1	5
5	SZGMW9	Small stream	Shenzhen, Guangming	22.7850, 113.9512	2019-04-26	LP	27	2	10
6	SZGMW11	Small stream	Shenzhen, Guangming	22.7840, 113.9515	2019-05-10	LP	26	4	20
7	SZGMW12	Small stream	Shenzhen, Guangming	22.7850, 113.9502	2019-05-10	LP$	26	10	50
8	SZGMW13	Small stream	Shenzhen, Guangming	22.7837, 113.9551	2019-05-10	LP$	26	17	85
9	SZGMW18	Small stream	Shenzhen, Guangming	22.7834, 113.9535	2019-05-10	LP$	26	1	5
10	SZGMW19	Small stream	Shenzhen, Guangming	22.7834, 113.9530	2019-05-10	LP	26	1	5
11	SZGMW20	Small stream	Shenzhen, Guangming	22.7834, 113.9527	2019-05-10	LP$	26	38	190
12	SZGMW26	Small stream	Shenzhen, Guangming	22.7824, 113.9614	2019-05-17	LS	29	3	15
13	SZGMW28	Small stream	Shenzhen, Guangming	22.7824, 113.9618	2019-05-17	LP$	29	12	60
14	SZGMW30	Small stream	Shenzhen, Guangming	22.7809, 113.9602	2019-05-17	LP$	29	2	10
15	GZBYW2	Pond	Guangzhou, Baiyun	23.1876, 113.3320	2019-05-12	LP	29	7	35
16	GZBYW5	Pond	Guangzhou, Baiyun	23.1880, 113.3322	2019-05-12	LP	29	1	5
17	GZBYW8	Pond	Guangzhou, Baiyun	23.1831, 113.3300	2019-05-12	LS	32	12	60
18	GZBYW9	Pond	Guangzhou, Baiyun	23.1829, 113.3298	2019-05-12	LS	32	2	10
19	GZBYW10	Small stream	Guangzhou, Baiyun	23.1829, 113.3298	2019-06-09	LP	32	11	55
20	GZBYW26	Small stream	Guangzhou, Baiyun	23.1847, 113.3382	2019-06-09	LP$	31	24	120
21	GZBYW27	Small stream	Guangzhou, Baiyun	23.1846, 113.3383	2019-06-09	LP$	31	5	25
22	GZBYW29	Small stream	Guangzhou, Baiyun	23.1843, 113.3383	2019-06-09	LP $/LG	31	2	10
23	HSXLW12	Lake	Huangshi, Xialu	30.1817, 115.0323	2019-06-13	LP	27	3	15
24	HSXLW13	Lake	Huangshi, Xialu	30.1819, 115.0327	2019-06-13	LP	27	29	145
25	HSXLW15	Fountain	Huangshi, Xialu	30.2020, 115.0435	2019-06-13	LP	27	10	50
26	HSXLW16	Fountain	Huangshi, Xialu	30.1986, 115.0410	2019-06-13	LP$	27	1	5
27	HSXLW20	Fountain	Huangshi, Xialu	30.1994, 115.0431	2019-06-13	LP$	28	1	5
28	HSXLW21	River	Huangshi, Xisanshan	30.2126, 115.0989	2019-06-14	LP/LG	28	32	160
29	QYYDW7	Pond	Qingyuan Yingde	23.3388, 113.3238	2019-06-29	LP$	33	5	25
30	QYYDW8	Pond	Qingyuan Yingde	24.3399, 113.3281	2019-06-29	LP	33	1	5
31	QYYDW9	Pond	Qingyuan, Yingde	24.3575, 113.3318	2019-06-29	LP	33	10	50
32	QYYDW11	River	Qingyuan, Yingde	24.2760, 113.3429	2019-06-29	LG	33	10	50
33	SZGMW34	Pond	Shenzhen, Guangming	22.8011, 113.9449	2019-07-04	LP$/LLo	31	8	40
34	SZGMW36	Pond	Shenzhen, Guangming	22.8011, 113.9449	2019-07-04	LP	31	17	85
35	SZGMW37	Pond	Shenzhen, Guangming	22.8046, 113.9463	2019-07-04	LLo	31	1	5
36	SZGMW39	Grassland puddle	Shenzhen, Guangming	22.8029, 113.9460	2019-07-04	LP$	31	28	140
37	SZGMW41	Lake	Shenzhen, Guangming	22.7986, 113.9544	2019-07-09	LP/LG	30	16	80
38	SZGMW42	Lake	Shenzhen, Guangming	22.7708, 113.9612	2019-07-09	LP$/LG	30	3	15
39	SZGMW50	Pond	Shenzhen, Guangming	22.7631, 113.9660	2019-07-09	LP	30	1	5
40	SZGMW51	Pond	Shenzhen, Guangming	22.7620, 113.9666	2019-07-09	LP$	30	4	20
41	SZGMW63	Lake	Shenzhen, Guangming	22.7680, 113.9465	2019-07-19	LP$/LG/LLo/LS	33	33	165
42	SZGMW57	Lake	Shenzhen, Guangming	22.7646, 113.9487	2019-07-19	LP$	33	1	5
43	NJPKW1	River	Nanjing, Pukou	32.1334, 118.7587	2019-07-28	LP$	34	4	20
44	NJPKW2	River	Nanjing, Pukou	32.1548, 118.8074	2019-07-28	LP$	34	1	5
45	NJPKW4	River	Nanjing, Pukou	32.1121, 118.7370	2019-07-28	LP$	34	11	55
46	NJPKW5	River	Nanjing, Pukou	22.7361, 113.8920	2019-07-28	LP$	34	19	95
47	SZGMW65	Lake	Shenzhen, Guangming	22.7377, 113.8916	2019-07-28	LP$/LG/LLo	30	27	135
48	SZGMW66	Lake	Shenzhen, Guangming	22.7377, 113.8916	2019-07-28	LP$/LLo	30	25	125
49	SZGMW68	Lake	Shenzhen, Guangming	22.7385, 113.8932	2019-07-28	LP	30	4	20
50	SZGMW69	Lake	Shenzhen, Guangming	22.7416, 113.8997	2019-07-28	LP$	30	5	25
51	SZGMW70	Lake	Shenzhen, Guangming	22.7386, 113.8942	2019-07-28	LP$	30	2	10
52	SZGMW72	Lake	Shenzhen, Guangming	22.7364, 113.8957	2019-07-28	LP$	30	4	20
53	SZGMW73	Lake	Shenzhen, Guangming	22.7372, 113.8995	2019-07-28	LP	30	18	90
54	SZGMW74	Lake	Shenzhen, Guangming	22.7401, 113.9004	2019-07-28	LP$	30	12	60
55	SZFTW76	Sea water	Shenzhen, Futian	22.5191, 113.9895	2019-08-04	LP/LLo	30	18	90
56	SZFTW77	Sea water	Shenzhen, Futian	22.5183, 113.9794	2019-08-04	LP$	30	20	100
57	SZFTW79	Sea water	Shenzhen, Futian	22.5182, 113.9797	2019-08-04	LP$	30	2	10
58	SZFTW80	Sea water	Shenzhen, Futian	22.5181, 113.9798	2019-08-04	LP$/LLo	30	20	100
59	SZFTW83	Lake	Shenzhen, Futian	22.5263, 113.9834	2019-08-04	LP$/LG	30	2	10
60	SZFTW84	Lake	Shenzhen, Futian	22.5266, 113.9834	2019-08-04	LP/LLo	30	18	90
61	SZFTW85	Lake	Shenzhen, Futian	22.5271, 113.9887	2019-08-04	LP/LLo/LG	30	14	70
62	DLSHKW7	River	Dalian, Shahekou	38.8882, 121.5946	2019-08-23	LP$	30	2	10
63	DLSHKW9	River	Dalian, Shahekou	38.8929, 121.5935	2019-08-23	LP	30	1	5
64	DGSSHW4	Lake	Dongguan, SOngshanhu	22.9224, 113.8947	2019-08-18	LP	30	1	5
65	CZLAW1	River	Chuzhou, Laian	32.2011, 118.6031	2019-08-18	LG	31	1	5
66	CZLAW2	River	Chuzhou, Laian	32.2022, 118.6052	2019-08-18	LLo	31	21	105
67	CZLAW3	River	Chuzhou, Laian	32.2004, 118.5969	2019-08-18	LLo	31	27	135
68	CZLAW4	River	Chuzhou, Laian	32.2048, 118.6150	2019-08-18	LLo/LF	31	17	85
69	CZLAW5	River	Chuzhou, Laian	32.1932, 118.5942	2019-08-18	LLo	31	19	95
70	GZYXW35	Lake	Guangzhou, Yuexiu	23.1452, 113.2623	2019-08-25	LP	29	37	185
71	GZYXW37	Lake	Guangzhou, Yuexiu	23.1448, 113.2621	2019-08-25	LP	29	7	35
72	GZYXW38	Lake	Guangzhou, Yuexiu	23.1434, 113.2620	2019-08-25	LP	29	71	355
73	GZYXW39	Lake	Guangzhou, Yuexiu	23.1389, 113.2635	2019-08-25	LP$	29	69	345
74	GZBYW40	Lake	Guangzhou,Baiyun	23.1569, 113.2940	2019-09-16	LP$	32	1	5
75	GZBYW43	Lake	Guangzhou, Baiyun	23.1572, 113.2940	2019-09-16	LP$	32	3	15
76	GZBYW44	Lake	Guangzhou, Baiyun	23.1573, 113.2935	2019-09-16	LP$/LM	32	4	20
77	GZBYW48	Grassland puddle	Guangzhou, Baiyun	23.1571, 113.2933	2019-09-16	LP$/LM	32	2	10
78	BJCYW4	Lake	Beijing, Chaoyang	39.9270, 116.4645	2019-09-13	LG	24	1	5
79	BJCYW11	River	Beijing, Chaoyang	39.9054, 116.4889	2019-09-13	LP/LM/LLo	24	9	45
80	BJCYW12	River	Beijing, Chaoyang	39.9043, 116.4827	2019-09-13	LP$/LS/LG/LLo	24	13	65
81	BJCYW13	River	Beijing, Chaoyang	39.9046, 116.4711	2019-09-13	LP/LG/LLo	24	17	85

82	BJCYW14	River	Beijing, Chaoyang	39.9040, 116.4658	2019-09-13	LP/LB	24	6	30
83	BJCYW15	River	Beijing, Chaoyang	39.9037, 116.4563	2019-09-13	LP	24	2	10
84	MZDPW5	Small stream	Meizhou, Dapu	24.4873, 116.7598	2019-10-02	LP	34	53	265
85	GZBYW49	Lake	Guangzhou, Baiyun	23.1885, 113.3324	2019-10-26	LP	25	28	140
86	GZBYW51	Lake	Guangzhou, Baiyun	23.1883, 113.3319	2019-10-26	LP	25	19	95
87	GZBYW52	Lake	Guangzhou, Baiyun	23.1878, 113.3324	2019-10-26	LP	25	16	80
88	GZBYW54	Lake	Guangzhou, Baiyun	23.1875, 113.3322	2019-10-26	LP	25	12	60
89	HZHYW3	Lake	Huizhou, Huiyang	22.7369, 114.5365	2019-10-31	LP$/LG	26	5	25
90	HZHYW4	Lake	Huizhou, Huiyang	22.7350, 114.5324	2019-10-31	LP$/LLo	26	7	35
91	HZHYW5	Lake	Huizhou, Huiyang	22.7353, 114.5367	2019-10-31	LP$	26	24	120
92	CDDJYW1	River	Chengdu, Dujiangyan	31.0010, 103.6111	2019-10-30	LP	16	2	10
93	CDJJW2	River	Chengdu, Jingjiang	30.6550, 104.0893	2019-10-30	LP/LG	16	57	285
94	CDJJW3	Pond	Chengdu, Jingjiang	30.6572, 104.0717	2019-10-30	LG	16	2	10
95	CDWHW5	Pond	Chengdu, Wuhou	30.6453, 104.0468	2019-10-30	LG	16	1	5
96	CQYZW1	River	Chongqing, Yuzhong	29.5604, 106.5401	2019-10-31	LG	14	1	5
97	SHXHW8	River	Shanghai, Xuhui	31.1642, 121.4180	2019-11-02	LLo	21	13	65
98	SZGMW86	Small stream	Shenzhen, Guangming	22.7849, 113.9491	2020-03-02	LG	23	2	10
99	SZGMW89	Small stream	Shenzhen, Guangming	22.7849, 113.9500	2020-03-02	LP$	23	1	5
100	SZGMW96	Small stream	Shenzhen, Guangming	22.7842, 113.9515	2020-03-02	LP$	23	4	20
101	NJQHW6	Small stream	Nanjing, Qinhuai	32.0245, 118.8015	2020-07-18	LG/LS/LLo	25	31	155
102	NJQHW7	Small stream	Nanjing, Qinhuai	32.0235, 118.8017	2020-07-18	LG/LS	25	3	15
103	NJQHW8	Small stream	Nanjing, Qinhuai	32.0270, 118.8977	2020-07-18	LG/LS/LLo/LM/LP	25	57	285
104	SZGMW97	Small stream	Shenzhen, Guangming	22.7679, 113.9460	2020-09-15	LP$/LDu	27	12	60
105	SZGMW98	Small stream	Shenzhen, Guangming	22.7681, 113.9458	2020-09-15	LP$	27	8	40
106	SZGMW99	Small stream	Shenzhen, Guangming	22.7673, 113.9462	2020-09-15	LP$	27	14	70
107	SZGMW100	Small stream	Shenzhen, Guangming	22.7685, 113.9454	2020-09-15	LP	27	2	10
108	SZGMW101	Small stream	Shenzhen, Guangming	22.7688, 113.9451	2020-09-15	LP/LDu	27	6	30
109	SZGMW102	Small stream	Shenzhen, Guangming	22.7687, 113.9453	2020-09-15	LP	27	1	5
110	SZGMW103	Small stream	Shenzhen, Guangming	22.7691, 113.9448	2020-09-15	LP$/LDu	27	28	140
111	SZGMW104	Small stream	Shenzhen, Guangming	22.7687, 113.9451	2020-09-15	LP$	27	7	35
112	SZGMW105	Small stream	Shenzhen, Guangming	22.7682, 113.9457	2020-09-15	LP/LG	27	4	20
113	SZGMW106	Small stream	Shenzhen, Guangming	22.7645, 113.9324	2020-09-22	LP$	29	12	60
114	SZGMW107	Small stream	Shenzhen, Guangming	22.7677, 113.9322	2020-09-22	LP$	29	11	55
115	SZGMW108	Small stream	Shenzhen, Guangming	22.7687, 113.9272	2020-09-22	LP$	29	7	35
116	SZGMW109	Small stream	Shenzhen, Guangming	22.7689, 113.9236	2020-09-22	LP	29	3	15
117	SZGMW110	Small stream	Shenzhen, Guangming	22.7705, 113.9205	2020-09-22	LP$	29	9	45
118	SZGMW111	Small stream	Shenzhen, Guangming	22.7712, 113.9183	2020-09-22	LP$	29	6	30
119	SZGMW112	Sea water	Shenzhen, Yantian	22.5946, 114.3117	2020-10-03	LC	30	4	20
120	SZGMW115	Pond	Shenzhen, Guangming	22.7613, 113.9707	2020-10-07	LP	25	3	15
121	SZGMW116	Small stream	Shenzhen, Guangming	22.7641, 113.9508	2020-10-20	LP	25	1	5
122	SZGMW117	Small stream	Shenzhen, Guangming	22.7644, 113.9512	2020-10-20	LP	25	1	5
123	SZGMW122	Small stream	Shenzhen, Guangming	22.7644, 113.9501	2020-10-20	LP$	25	5	25
124	SZGMW123	Small stream	Shenzhen, Guangming	22.7643, 113.9494	2020-10-20	LP	25	4	20
125	GZYXW55	Lake	Guangzhou, Yuexiu	23.1355, 113.2508	2020-11-21	LP$/LDu/LLo	25	5	25
126	SZGMW127	Small stream	Shenzhen, Guangming	22.7846, 113.9519	2021-02-22	LP	25	2	10
127	SZGMW129	Small stream	Shenzhen, Guangming	22.7845, 113.9500	2021-02-22	LP$	27	63	315
128	SZGMW130	Small stream	Shenzhen, Guangming	22.7852, 113.9534	2021-02-22	LP	27	1	5
129	SZGMW131	Small stream	Shenzhen, Guangming	22.7847. 113.9502	2021-02-22	LP$	27	2	10
Soil samples	Soil isolates								
1	QDNHPS4	Garden soil	Qiandongnan, Huangping	27.0120, 107.7938	2019-10-02	LMo	28	3	300
2	SJZZDS4	Garden soil	Shijiazhuang, Zhengding	38.1376, 114.5598	2019-10-17	LP$/LF/LO	13	12	1200
3	CQYZS2	Garden soil	Chongqing, Yuzhong	29.5619, 106.5779	2019-10-30	LB	15	1	100
4	GZHZS8	Potted soil	Guangzhou, Haizhu	23.0760, 113.3218	2019-11-03	LP/LM/LLo	27	5	500
5	GZHZS9	Potted soil	Guangzhou, Haizhu	23.0726, 113.3194	2019-11-03	LP$	27	15	1500
6	GZHZS11	Potted soil	Guangzhou, Haizhu	23.1542, 113.2717	2019-11-17	LM	26	1	100
7	GZBYS13	Potted soil	Guangzhou, Haizhu	23.1505, 113.2682	2019-11-17	LM	26	1	100
8	GZBYS17	Garden soil	Guangzhou, Baiyun	23.1477, 113.2709	2019-11-17	LP	26	3	300
9	GZBYS19	Potted soil	Guangzhou, Baiyun	23.1496, 113.2699	2019-11-17	LDu/LM/LLo	26	25	2500
10	GZTHS22	Garden soil	Guangzhou, Tianhe	23.1845, 113.3433	2019-11-24	LM	23	2	200
11	GZBYS23	Garden soil	Guangzhou, Baiyun	23.1926, 113.3071	2019-11-24	LP$	23	14	1400
12	GZTHS24	Garden soil	Guangzhou, Tianhe	23.1849, 113.3432	2019-11-24	LP	23	1	100
13	GZYXS29	Potted soil	Guangzhou, Yuexiu	23.1394, 113.2951	2019-12-01	LP	22	1	100
14	GZYXS33	Potted soil	Guangzhou, Yuexiu	23.1403, 113.2961	2019-12-01	LM	22	1	100
15	GZYXS37	Potted soil	Guangzhou, Yuexiu	23.1410, 113.2941	2019-12-01	LP	22	1	100
16	GZYXS43	Potted soil	Guangzhou, Yuexiu	23.1787, 113.3212	2019-12-08	LM	18	1	100
17	GZBYS47	Garden soil	Guangzhou, Baiyun	23.1788, 113.3210	2019-12-08	LDu/LP	18	113	11300
18	GZBYS51	Potted soil	Guangzhou, Baiyun	23.1815, 113.3204	2019-12-08	LDu/LP	18	15	1500
19	GZBYS52	Potted soil	Guangzhou, Baiyun	23.1813, 113.3199	2019-12-08	LP/LM	18	3	300
20	GZBYS53	Potted soil	Guangzhou, Baiyun	23.1813, 113.3205	2019-12-08	LP	18	2	200
21	SZGMS1	Garden soil	Shenzhen, Guangming	22.7853, 113.9500	2019-12-12	LP$	21	14	1400
22	SZGMS2	Garden soil	Shenzhen, Guangming	22.7853, 113.9498	2019-12-12	LP	21	18	1800
23	GZBYS61	Garden soil	Guangzhou, Baiyun	23.1828, 113.3345	2019-12-27	LP$	19	2	200
24	GZBYS64	Garden soil	Guangzhou, Baiyun	23.1875, 113.3369	2019-12-27	LP	19	1	100
25	GZBYS66	Garden soil	Guangzhou, Baiyun	23.1873, 113.3368	2019-12-27	LP	19	1	100
26	SZGMS3	Garden soil	Shenzhen, Guangming	22.7854, 113.9499	2020-01-07	LP	25	3	300
27	SZGMS4	Garden soil	Shenzhen, Guangming	22.7852, 113.9501	2020-01-07	LP$/LDu	25	43	4300
28	SZGMS5	Garden soil	Shenzhen, Guangming	22.7854, 113.9496	2020-01-07	LP$	25	5	500
29	SZGMS6	Garden soil	Shenzhen, Guangming	22.7853, 113.9494	2020-01-07	LP$	25	36	3600
30	SZGMS7	Garden soil	Shenzhen, Guangming	22.7858, 113.9497	2020-01-07	LP	25	1	100
31	SZGMS8	Garden soil	Shenzhen, Guangming	22.7851, 113.9495	2020-01-07	LP	25	1	100
32	SZGMS9	Garden soil	Shenzhen, Guangming	22.7855, 113.9495	2020-01-07	LP$	25	23	2300
33	GZBYS74	Garden soil	Shenzhen, Guangming	23.1977, 113.3379	2020-01-11	LP	22	1	100
34	HSXSSS14	Garden soil	Huangshi, Xisanshan	30.1409, 115.0493	2020-04-09	LP/LM	25	49	4900

35	SZGMS16	Garden soil	Shenzhen, Guangming	22.7888, 113.9667	2020-05-08	LP$/LLo	31	44	4400
36	NJQHS1	Garden soil	Nanjing, Qinhuai	32.0239, 118.8018	2020-07-18	LM	25	1	100
37	NJQHS2	Garden soil	Nanjing, Qinhuai	32.0284, 118.8009	2020-07-18	LW	25	1	100
38	NJQHS3	Garden soil	Nanjing, Qinhuai	32.0277, 118.7995	2020-07-18	LG	25	2	200
39	GZZCS67	Garden soil	Guangzhou, Zengcheng	23.2386, 113.7827	2020-10-02	LP	30	1	100
40	GZZCS68	Garden soil	Guangzhou, Zengcheng	23.2417,113.8308	2020-10-02	LP$/LDu/LC/LG	30	24	2400
41	GZYXS72	Garden soil	Guangzhou, Zengcheng	23.1386, 113.2547	2021-01-16	LC	20	1	100

**LP: L. pneumophila; LG: L. gormanii; LLo: L. longbeachae; LS: L. sainthelensi, LM: L. micdadei, LB: L. bozemanae, LMo: L. moravica; LW: L. wadsworthii; LDu: L. dumoffii; LF: L. feeleii; LC: L*

$ indicates sg1 of L. pneumophila was found in these samples.

Table S2. ***L. pneumophila*** **ST distribution in the two sources of China.**

Source and number of isolates (n)	Sequence types (STs)	Shared STs	Private STs	Singletons of STs	ST diversity	Nucleotide diversity*
Water (263)	110	15	95	73	0.9789	0.03997
Soil (208)	82	15	67	47	0.9780	0.03961

* Nucleotide diversity was evaluated by using concatenated SBT sequences.

Table S3. SBT profiles and detailed information of 471 L. pneumophila isolates.

	Allelic profiles									Sample information of the isolates		
Isolate name	*flaA*	*pilE*	*asd*	*mip*	*mompS*	*proA*	*neuA/neuAh*	ST*	Temporary designation	Isolate city & Regione	Isolate time (yyyy-mm-dd	Source of sample
GZBYW26.1	1	4	3	1	1	1	1	1	N/A	Guangzhou, Baiyun	2019-06-09	Water: Small stream
GZBYW26.10	1	4	3	1	1	1	1	1	N/A	Guangzhou, Baiyun	2019-06-09	Water: Small stream
GZBYW26.20	1	4	3	1	1	1	1	1	N/A	Guangzhou, Baiyun	2019-06-09	Water: Small stream
GZBYW27.1	1	4	3	1	1	1	1	1	N/A	Guangzhou, Baiyun	2019-06-09	Water: Small stream
GZBYW27.5	1	4	3	1	1	1	1	1	N/A	Guangzhou, Baiyun	2019-06-09	Water: Small stream
GZBYW29.1	1	4	3	1	1	1	1	1	N/A	Guangzhou, Baiyun	2019-06-09	Water: Small stream
SZGMS2.9	12	9	26	5	26	17	15	15	N/A	Shenzhen, Guangming	2019-12-12	Soil: Garden soil
GZYXW39.54	2	10	9	13	2	5	6	18	N/A	Guangzhou, Yuexiu	2019-08-25	Water: Lake
SZFTW77.15	2	10	9	13	2	5	6	18	N/A	Shenzhen, Futian	2019-08-04	Water: Sea
SZGMW39.26	2	3	6	10	2	1	6	22	N/A	Shenzhen, Guangming	2019-07-04	Water: Grassland puddle
SZGMW66.2	2	3	6	10	2	1	6	22	N/A	Shenzhen, Guangming	2019-07-28	Water: Lake
SZGMW66.24	2	3	6	10	2	1	6	22	N/A	Shenzhen, Guangming	2019-07-28	Water: Lake
GZBYS47.54	5	1	22	26	6	10	12	45	N/A	Guangzhou, Baiyun	2019-12-08	Soil: Garden soil
GZBYS47.55	5	1	22	26	6	10	12	45	N/A	Guangzhou, Baiyun	2019-12-08	Soil: Garden soil
GZBYS47.99	5	1	22	26	6	10	12	45	N/A	Guangzhou, Baiyun	2019-12-08	Soil: Garden soil
NJPKW4.2	5	1	22	26	6	10	12	45	N/A	Nanjing, Pukou	2019-07-28	Water: River
NJPKW5.5	5	1	22	26	6	10	12	45	N/A	Nanjing, Pukou	2019-07-28	Water: River
SJZZDS4.6	5	1	22	26	6	10	12	45	N/A	Shijiazhuang, Zhengding	2019-10-17	Soil: Garden soil
SZFTW77.8	5	1	22	26	6	10	12	45	N/A	Shenzhen, Futian	2019-08-04	Water: Sea
SZGMW5.1	5	1	22	26	6	10	12	45	N/A	Shenzhen, Guangming	2019-04-26	Water: Small stream
SZGMW69.4	5	1	22	26	6	10	12	45	N/A	Shenzhen, Guangming	2019-07-28	Water: Lake
GZBYS47.103	5	2	22	27	6	10	12	48	N/A	Guangzhou, Baiyun	2019-12-08	Soil: Garden soil
GZBYS47.51	5	2	22	27	6	10	12	48	N/A	Guangzhou, Baiyun	2019-12-08	Soil: Garden soil
GZBYS47.59	5	2	22	27	6	10	12	48	N/A	Guangzhou, Baiyun	2019-12-08	Soil: Garden soil
GZBYS47.72	5	2	22	27	6	10	12	48	N/A	Guangzhou, Baiyun	2019-12-08	Soil: Garden soil
GZBYS47.88	5	2	22	27	6	10	12	48	N/A	Guangzhou, Baiyun	2019-12-08	Soil: Garden soil
GZBYS47.89	5	2	22	27	6	10	12	48	N/A	Guangzhou, Baiyun	2019-12-08	Soil: Garden soil
GZBYS47.91	5	2	22	27	6	10	12	48	N/A	Guangzhou, Baiyun	2019-12-08	Soil: Garden soil
HZHYW5.1	5	2	22	27	6	10	12	48	N/A	Huizhou, Huiyang	2019-10-31	Water: Lake
NJPKW1.2	5	2	22	27	6	10	12	48	N/A	Nanjing, Pukou	2019-07-28	Water: River
NJPKW4.6	5	2	22	27	6	10	12	48	N/A	Nanjing, Pukou	2019-07-28	Water: River
SZFTW77.20	5	2	22	27	6	10	12	48	N/A	Shenzhen, Futian	2019-08-04	Water: Sea
SZFTW77.5	5	2	22	27	6	10	12	48	N/A	Shenzhen, Futian	2019-08-04	Water: Sea
SZGMW18.1	5	2	22	27	6	10	12	48	N/A	Shenzhen, Guangming	2019-05-10	Water: Small stream
SZGMW28.1	5	2	22	27	6	10	12	48	N/A	Shenzhen, Guangming	2019-05-17	Water: Pond
SZGMW65.10	5	2	22	27	6	10	12	48	N/A	Shenzhen, Guangming	2019-07-28	Water: Lake
SZGMW74.11	5	2	22	27	6	10	12	48	N/A	Shenzhen, Guangming	2019-07-28	Water: Lake
SZGMW74.6	5	2	22	27	6	10	12	48	N/A	Shenzhen, Guangming	2019-07-28	Water: Lake
SZGMW96.3	5	2	22	27	6	10	12	48	N/A	Shenzhen, Guangming	2020-03-02	Water: Small stream
SZGMW122.1	5	2	22	27	6	10	12	48	N/A	Shenzhen, Guangming	2020-10-20	Water: Small stream
SZGMW13.1	5	1	22	30	6	10	6	74	N/A	Shenzhen, Guangming	2019-05-10	Water: Small stream
SZGMW13.15	5	1	22	30	6	10	6	74	N/A	Shenzhen, Guangming	2019-05-10	Water: Small stream
SZGMW28.8	5	1	22	30	6	10	6	74	N/A	Shenzhen, Guangming	2019-05-17	Water: Pond
SZGMW4.10	5	1	22	30	6	10	6	74	N/A	Shenzhen, Guangming	2019-04-26	Water: Small stream
SZGMS4.14	12	9	2	5	3	17	15	84	N/A	Shenzhen, Guangming	2020-01-07	Soil: Garden soil
SZGMS5.2	12	9	2	5	3	17	15	84	N/A	Shenzhen, Guangming	2020-01-07	Soil: Garden soil
SZGMS6.10	12	9	2	5	3	17	15	84	N/A	Shenzhen, Guangming	2020-01-07	Soil: Garden soil
SZGMS6.23	12	9	2	5	3	17	15	84	N/A	Shenzhen, Guangming	2020-01-07	Soil: Garden soil
SZGMS6.30	12	9	2	5	3	17	15	84	N/A	Shenzhen, Guangming	2020-01-07	Soil: Garden soil
SZGMS6.9	12	9	2	5	3	17	15	84	N/A	Shenzhen, Guangming	2020-01-07	Soil: Garden soil
GZBYS23.13	2	3	9	13	2	1	6	88	N/A	Guangzhou, Baiyun	2019-11-24	Soil: Garden soil
GZBYS23.14	2	3	9	13	2	1	6	88	N/A	Guangzhou, Baiyun	2019-11-24	Soil: Garden soil
GZBYS23.3	2	3	9	13	2	1	6	88	N/A	Guangzhou, Baiyun	2019-11-24	Soil: Garden soil
GZBYS23.4	2	3	9	13	2	1	6	88	N/A	Guangzhou, Baiyun	2019-11-24	Soil: Garden soil
HZHYW5.24	12	29	2	5	3	17	15	115	N/A	Huizhou, Huiyang	2019-10-31	Water: Lake
SZGMS6.16	12	29	2	5	3	17	15	115	N/A	Shenzhen, Guangming	2020-01-07	Soil: Garden soil
SZGMS6.27	12	29	2	5	3	17	15	115	N/A	Shenzhen, Guangming	2020-01-07	Soil: Garden soil
SZGMS6.28	12	29	2	5	3	17	15	115	N/A	Shenzhen, Guangming	2020-01-07	Soil: Garden soil
CDDJYW1.1	3	10	1	28	1	9	3	242	N/A	Chengdu, Dujiangyan	2019-10-30	Water: Lake
GZHZS9.6	12	8	11	23	29	26	2	260	N/A	Guangzhou, Haizhu	2019-11-03	Soil: Potted soil
GZYXW35.69	12	8	11	23	29	26	2	260	N/A	Guangzhou, Yuexiu	2019-08-25	Water: Lake
HSXSSS14.15	12	8	11	23	29	26	2	260	N/A	Huangshi, Xisaishan	2020-04-09	Soil: Garden soil
HSXSSS14.16	12	8	11	23	29	26	2	260	N/A	Huangshi, Xisaishan	2020-04-09	Soil: Garden soil
HSXSSS14.47	12	8	11	23	29	26	2	260	N/A	Huangshi, Xisaishan	2020-04-09	Soil: Garden soil
HSXSSS14.6	12	8	11	23	29	26	2	260	N/A	Huangshi, Xisaishan	2020-04-09	Soil: Garden soil
HZHYW5.14	12	8	11	23	29	26	2	260	N/A	Huizhou, Huiyang	2019-10-31	Water: Lake
NJPKW5.1	12	8	11	23	29	26	2	260	N/A	Nanjing, Pukou	2019-07-28	Water: River
NJPKW5.11	12	8	11	23	29	26	2	260	N/A	Nanjing, Pukou	2019-07-28	Water: River
SZGMS6.4	12	8	11	23	29	26	2	260	N/A	Shenzhen, Guangming	2020-01-07	Soil: Garden soil
SZGMS9.19	12	8	11	23	29	26	2	260	N/A	Shenzhen, Guangming	2020-01-07	Soil: Garden soil
SZGMW51.1	12	8	11	23	29	26	2	260	N/A	Shenzhen, Guangming	2019-07-09	Water: Pond
SZGMW63.23	12	8	11	23	29	26	2	260	N/A	Shenzhen, Guangming	2019-07-19	Water: Lake
SZGMW63.27	12	8	11	23	29	26	2	260	N/A	Shenzhen, Guangming	2019-07-19	Water: Lake
GZHZS9.11	7	10	17	3	13	11	11	269	N/A	Guangzhou, Haizhu	2019-11-03	Soil: Potted soil
SZGMW69.1	2	10	9	10	2	1	6	299	N/A	Shenzhen, Guangming	2019-07-28	Water: Lake
DLSHKW7.1	6	10	19	3	19	4	11	345	N/A	Daslian, Shihekou	2019-08-23	Water: River
SZGMW34.3	6	10	19	28	19	4	13	362	N/A	Shenzhen, Guangming	2019-07-04	Water: Pond
HZHYW5.13	6	10	15	28	21	14	9	367	N/A	Huizhou, Huiyang	2019-10-31	Water: Lake
HZHYW3.1	2	3	9	10	2	1	10	384	N/A	Huizhou, Huiyang	2019-10-31	Water: Lake
HZHYW3.5	2	3	9	10	2	1	10	384	N/A	Huizhou, Huiyang	2019-10-31	Water: Lake
HZHYW4.3	2	3	9	10	2	1	10	384	N/A	Huizhou, Huiyang	2019-10-31	Water: Lake
BJCYW14.2	6	10	15	3	21	14	9	407	N/A	Beijing, Chaoyang	2019-09-13	Water: River
GZBYS13.1	6	10	14	28	21	14	9	461	N/A	Guangzhou, Baiyun	2019-11-17	Soil: Garden soil
GZBYS17.1	6	10	14	28	21	14	9	461	N/A	Guangzhou, Baiyun	2019-11-17	Soil: Garden soil
SZGMS4.37	6	10	14	28	21	14	9	461	N/A	Shenzhen, Guangming	2020-01-07	Soil: Garden soil
SZGMS4.40	6	10	14	28	21	14	9	461	N/A	Shenzhen, Guangming	2020-01-07	Soil: Garden soil
SZGMW39.5	2	3	6	13	2	1	6	506	N/A	Shenzhen, Guangming	2019-07-04	Water: Grassland puddle
SZGMW65.25	3	13	1	3	14	9	11	579	N/A	Shenzhen, Guangming	2019-07-28	Water: Lake
HSXSSS14.12	17	23	13	20	32	22	205	707	N/A	Huangshi, Xisaishan	2020-04-09	Soil: Garden soil
HSXSSS14.18	17	23	13	20	32	22	205	707	N/A	Huangshi, Xisaishan	2020-04-09	Soil: Garden soil
HSXSSS14.24	17	23	13	20	32	22	205	707	N/A	Huangshi, Xisaishan	2020-04-09	Soil: Garden soil
HSXSSS14.9	17	23	13	20	32	22	205	707	N/A	Huangshi, Xisaishan	2020-04-09	Soil: Garden soil
HSXSSS14.1	12	29	2	5	50	20	15	710	N/A	Huangshi, Xisaishan	2020-04-09	Soil: Garden soil
HSXSSS14.23	12	29	2	5	50	20	15	710	N/A	Huangshi, Xisaishan	2020-04-09	Soil: Garden soil
HSXSSS14.30	12	29	2	5	50	20	15	710	N/A	Huangshi, Xisaishan	2020-04-09	Soil: Garden soil

HSXSSS14.32	12	29	2	5	50	20	15	710	N/A	Huangshi, Xisaishan	2020-04-09	Soil: Garden soil
HSXSSS14.36	12	29	2	5	50	20	15	710	N/A	Huangshi, Xisaishan	2020-04-09	Soil: Garden soil
HSXSSS14.39	12	29	2	5	50	20	15	710	N/A	Huangshi, Xisaishan	2020-04-09	Soil: Garden soil
HSXSSS14.43	12	29	2	5	50	20	15	710	N/A	Huangshi, Xisaishan	2020-04-09	Soil: Garden soil
HSXSSS14.45	12	29	2	5	50	20	15	710	N/A	Huangshi, Xisaishan	2020-04-09	Soil: Garden soil
HSXSSS14.8	12	29	2	5	50	20	15	710	N/A	Huangshi, Xisaishan	2020-04-09	Soil: Garden soil
GZBYS23.5	12	8	11	2	10	12	2	739	N/A	Guangzhou, Baiyun	2019-11-24	Soil: Garden soil
SZGMW51.4	12	8	11	2	10	12	2	739	N/A	Shenzhen, Guangming	2019-07-09	Water: Pond
SZGMW96.1	12	8	11	2	10	12	2	739	N/A	Shenzhen, Guangming	2020-03-02	Water: Small stream
NJPKW5.10	2	3	9	15	2	1	6	758	N/A	Nanjing, Pukou	2019-07-28	Water: River
SZGMW41.4	6	10	19	28	19	4	11	763	N/A	Shenzhen, Guangming	2019-07-09	Water: Lake
SZGMW42.3	6	10	19	28	19	4	11	763	N/A	Shenzhen, Guangming	2019-07-09	Water: Lake
SZGMW98.1	2	3	9	10	2	6	6	995	N/A	Shenzhen, Guangming	2020-09-15	Water: Small stream
SZGMW102.1	2	3	9	10	2	6	6	995	N/A	Shenzhen, Guangming	2020-09-15	Water: Small stream
GZBYS47.102	2	3	9	10	2	6	6	995	N/A	Guangzhou, Baiyun	2019-12-08	Soil: Garden soil
GZBYS47.23	2	3	9	10	2	6	6	995	N/A	Guangzhou, Baiyun	2019-12-08	Soil: Garden soil
GZBYS47.25	2	3	9	10	2	6	6	995	N/A	Guangzhou, Baiyun	2019-12-08	Soil: Garden soil
GZBYS47.43	2	3	9	10	2	6	6	995	N/A	Guangzhou, Baiyun	2019-12-08	Soil: Garden soil
NJPKW4.1	2	3	9	10	2	6	6	995	N/A	Nanjing, Pukou	2019-07-28	Water: River
NJPKW4.10	2	3	9	10	2	6	6	995	N/A	Nanjing, Pukou	2019-07-28	Water: River
SZGMS4.6	2	3	9	10	2	6	6	995	N/A	Shenzhen, Guangming	2020-01-07	Soil: Garden soil
SZGMS4.9	2	3	9	10	2	6	6	995	N/A	Shenzhen, Guangming	2020-01-07	Soil: Garden soil
SZGMW2.10	2	3	9	10	2	6	6	995	N/A	Shenzhen, Guangming	2019-04-26	Water: Small stream
SZGMW2.5	2	3	9	10	2	6	6	995	N/A	Shenzhen, Guangming	2019-04-26	Water: Small stream
CDJJW2.37	3	13	1	6	14	9	38	1032	N/A	Chengdu, Dujiangyan	2019-10-30	Water: River
CDJJW2.43	3	13	1	6	14	9	38	1032	N/A	Chengdu, Dujiangyan	2019-10-30	Water: River
HZHYW4.7	2	10	14	10	21	4	3	1119	N/A	Huizhou, Huiyang	2019-10-31	Water: Lake
SZGMW42.1	2	15	9	10	2	1	6	1248	N/A	Shenzhen, Guangming	2019-07-09	Water: Lake
SZGMW103.20	5	1	22	30	6	10	203	1324	N/A	Shenzhen, Guangming	2020-09-15	Water: Small stream
SZGMW122.3	5	1	22	30	6	10	203	1324	N/A	Shenzhen, Guangming	2020-10-20	Water: Small stream
SZGMW123.1	5	1	22	30	6	10	203	1324	N/A	Shenzhen, Guangming	2020-10-20	Water: Small stream
GZBYS47.26	5	1	22	30	6	10	203	1324	N/A	Guangzhou, Baiyun	2019-12-08	Soil: Garden soil
GZBYS47.34	5	1	22	30	6	10	203	1324	N/A	Guangzhou, Baiyun	2019-12-08	Soil: Garden soil
GZBYS47.36	5	1	22	30	6	10	203	1324	N/A	Guangzhou, Baiyun	2019-12-08	Soil: Garden soil
GZBYS47.63	5	1	22	30	6	10	203	1324	N/A	Guangzhou, Baiyun	2019-12-08	Soil: Garden soil
GZBYS47.80	5	1	22	30	6	10	203	1324	N/A	Guangzhou, Baiyun	2019-12-08	Soil: Garden soil
GZBYS66.1	5	1	22	30	6	10	203	1324	N/A	Guangzhou, Baiyun	2019-12-27	Soil: Garden soil
HZHYW5.5	5	1	22	30	6	10	203	1324	N/A	Huizhou, Huiyang	2019-10-31	Water: Lake
SZFTW77.12	5	1	22	30	6	10	203	1324	N/A	Shenzhen, Futian	2019-08-04	Water: Sea
SZFTW77.6	5	1	22	30	6	10	203	1324	N/A	Shenzhen, Futian	2019-08-04	Water: Sea
SZGMS9.12	5	1	22	30	6	10	203	1324	N/A	Shenzhen, Guangming	2020-01-07	Soil: Garden soil
SZGMS9.17	5	1	22	30	6	10	203	1324	N/A	Shenzhen, Guangming	2020-01-07	Soil: Garden soil
SZGMS9.21	5	1	22	30	6	10	203	1324	N/A	Shenzhen, Guangming	2020-01-07	Soil: Garden soil
SZGMS9.6	5	1	22	30	6	10	203	1324	N/A	Shenzhen, Guangming	2020-01-07	Soil: Garden soil
SZGMW12.9	5	1	22	30	6	10	203	1324	N/A	Shenzhen, Guangming	2019-05-10	Water: Small stream
SZGMW4.2	5	1	22	30	6	10	203	1324	N/A	Shenzhen, Guangming	2019-04-26	Water: Small stream
SZGMS4.23	12	8	11	20	40	12	216	1332	N/A	Shenzhen, Guangming	2020-01-07	Soil: Garden soil
SZGMW110.1	6	10	14	28	21	14	207	1351	N/A	Shenzhen, Guangming	2020-09-22	Water: Small stream
GZBYS51.11	6	10	14	28	21	14	207	1351	N/A	Guangzhou, Baiyun	2019-12-08	Soil: Potted soil
GZBYS51.2	6	10	14	28	21	14	207	1351	N/A	Guangzhou, Baiyun	2019-12-08	Soil: Potted soil
GZBYS51.5	6	10	14	28	21	14	207	1351	N/A	Guangzhou, Baiyun	2019-12-08	Soil: Potted soil
GZBYS51.8	6	10	14	28	21	14	207	1351	N/A	Guangzhou, Baiyun	2019-12-08	Soil: Potted soil
GZBYS52.1	6	10	14	28	21	14	207	1351	N/A	Guangzhou, Baiyun	2019-12-08	Soil: Potted soil
GZBYS47.62	5	2	22	10	6	25	203	1358	N/A	Guangzhou, Baiyun	2019-12-08	Soil: Garden soil
HSXLW15.1	6	10	15	28	21	7	207	1439	N/A	Huangshi, Xialu	2019-06-13	Water: Fountain
GZYXW39.2	12	8	11	21	40	12	9	1694	N/A	Guangzhou, Yuexiu	2019-08-25	Water: Lake
GZYXW39.42	12	8	11	21	40	12	9	1694	N/A	Guangzhou, Yuexiu	2019-08-25	Water: Lake
GZYXW39.44	12	8	11	21	40	12	9	1694	N/A	Guangzhou, Yuexiu	2019-08-25	Water: Lake
HSXSSS14.26	12	8	11	21	40	12	9	1694	N/A	Huangshi, Xisaishan	2020-04-09	Soil: Garden soil
HSXSSS14.37	12	8	11	21	40	12	9	1694	N/A	Huangshi, Xisaishan	2020-04-09	Soil: Garden soil
SZFTW79.2	12	8	11	21	40	12	9	1694	N/A	Shenzhen, Futian	2019-08-04	Water: Sea
SZFTW80.1	12	8	11	21	40	12	9	1694	N/A	Shenzhen, Futian	2019-08-04	Water: Sea
SZFTW80.10	12	8	11	21	40	12	9	1694	N/A	Shenzhen, Futian	2019-08-04	Water: Sea
SZFTW80.16	12	8	11	21	40	12	9	1694	N/A	Shenzhen, Futian	2019-08-04	Water: Sea
SZFTW80.17	12	8	11	21	40	12	9	1694	N/A	Shenzhen, Futian	2019-08-04	Water: Sea
SZFTW80.20	12	8	11	21	40	12	9	1694	N/A	Shenzhen, Futian	2019-08-04	Water: Sea
SZGMW39.6	12	8	11	21	40	12	9	1694	N/A	Shenzhen, Guangming	2019-07-04	Water: Grassland puddle
SZGMW66.10	12	8	11	21	40	12	9	1694	N/A	Shenzhen, Guangming	2019-07-28	Water: Lake
GZBYS47.110	5	1	22	27	6	10	12	1707	N/A	Guangzhou, Baiyun	2019-12-08	Soil: Garden soil
GZYXW39.45	27	3	9	12	2	1	6	1712	N/A	Guangzhou, Yuexiu	2019-08-25	Water: Lake
SZFTW77.1	27	3	9	12	2	1	6	1712	N/A	Shenzhen, Futian	2019-08-04	Water: Sea
SZGMS6.18	27	3	9	12	2	1	6	1712	N/A	Shenzhen, Guangming	2020-01-07	Soil: Garden soil
SZGMS6.19	27	3	9	12	2	1	6	1712	N/A	Shenzhen, Guangming	2020-01-07	Soil: Garden soil
SZGMS6.21	27	3	9	12	2	1	6	1712	N/A	Shenzhen, Guangming	2020-01-07	Soil: Garden soil
SZGMS6.25	27	3	9	12	2	1	6	1712	N/A	Shenzhen, Guangming	2020-01-07	Soil: Garden soil
SZGMS6.36	27	3	9	12	2	1	6	1712	N/A	Shenzhen, Guangming	2020-01-07	Soil: Garden soil
SZGMW39.27	27	3	9	12	2	1	6	1712	N/A	Shenzhen, Guangming	2019-07-04	Water: Grassland puddle
SZGMW65.1	27	3	9	12	2	1	6	1712	N/A	Shenzhen, Guangming	2019-07-28	Water: Lake
SZGMW72.1	27	3	9	12	2	1	6	1712	N/A	Shenzhen, Guangming	2019-07-28	Water: Lake
SZGMW73.13	27	3	9	12	2	1	6	1712	N/A	Shenzhen, Guangming	2019-07-28	Water: River
GZBYS47.70	2	3	40	69	87	5	6	1731	N/A	Guangzhou, Baiyun	2019-12-08	Soil: Garden soil
SZGMS4.34	12	29	2	5	50	20	213	1889	N/A	Shenzhen, Guangming	2020-01-07	Soil: Garden soil
SZGMW108.1	2	10	9	10	2	1	229	1959	N/A	Shenzhen, Guangming	2020-09-22	Water: Small stream
GZYXW35.3	2	10	9	10	2	1	229	1959	N/A	Guangzhou, Yuexiu	2019-08-25	Water: Lake
NJPKW1.1	2	10	9	10	2	1	229	1959	N/A	Nanjing, Pukou	2019-07-28	Water: River
SZFTW80.2	2	10	9	10	2	1	229	1959	N/A	Shenzhen, Futian	2019-08-04	Water: Sea
SZFTW80.9	2	10	9	10	2	1	229	1959	N/A	Shenzhen, Futian	2019-08-04	Water: Sea
SZGMW66.1	2	10	9	10	2	1	229	1959	N/A	Shenzhen, Guangming	2019-07-28	Water: Lake
SZGMS4.31	12	9	2	21	3	20	15	2142	N/A	Shenzhen, Guangming	2020-01-07	Soil: Garden soil
GZYXW39.20	27	3	9	43	56	1	3	2154	N/A	Guangzhou, Yuexiu	2019-08-25	Water: Lake
GZYXW39.30	27	3	9	43	56	1	3	2154	N/A	Guangzhou, Yuexiu	2019-08-25	Water: Lake
SZGMW100.1	6	10	5	3	4	14	9	2246	N/A	Shenzhen, Guangming	2020-09-15	Water: Small stream
SZGMS1.8	6	10	5	3	4	14	9	2246	N/A	Shenzhen, Guangming	2019-12-12	Soil: Garden soil
SZGMS2.1	6	10	5	3	4	14	9	2246	N/A	Shenzhen, Guangming	2019-12-12	Soil: Garden soil
HSXSSS14.11	4	8	11	23	11	12	37	2437	N/A	Huangshi, Xisaishan	2020-04-09	Soil: Garden soil
NJPKW5.18	4	8	11	23	11	12	37	2437	N/A	Nanjing, Pukou	2019-07-28	Water: River
SZGMW20.1	4	8	11	23	11	12	37	2437	N/A	Shenzhen, Guangming	2019-05-10	Water: Small stream
SZGMW20.10	4	8	11	23	11	12	37	2437	N/A	Shenzhen, Guangming	2019-05-10	Water: Small stream

SZGMW20.20	4	8	11	23	11	12	37	2437	N/A	Shenzhen, Guangming	2019-05-10	Water: Small stream
SZGMW20.30	4	8	11	23	11	12	37	2437	N/A	Shenzhen, Guangming	2019-05-10	Water: Small stream
SZGMW20.38	4	8	11	23	11	12	37	2437	N/A	Shenzhen, Guangming	2019-05-10	Water: Small stream
SZGMW30.1	4	8	11	23	11	12	37	2437	N/A	Shenzhen, Guangming	2019-05-17	Water: Pond
NJPKW5.2	5	1	22	5	6	10	203	2584	N/A	Nanjing, Pukou	2019-07-28	Water: River
SJZZDS4.3	5	1	22	5	6	10	203	2584	N/A	Shijiazhuang, Zhengding	2019-10-17	Soil: Garden soil
SZGMS1.4	6	10	14	3	2	14	9	2661	N/A	Shenzhen, Guangming	2019-12-12	Soil: Garden soil
GZBYS74.1	2	12	48	6	48	15	220	2796	N/A	Guangzhou, Baiyun	2020-01-11	Soil: Garden soil
GZHZS9.9	12	8	11	23	201#	26	301	N/A	C1	Guangzhou, Haizhu	2019-11-03	Soil: Potted soil
QYYDW9.10	8	6	34	9	2	50	302	N/A	C2	Qingyuan, Yingde	2019-06-29	Water: Pond
BJCYW12.2	8	6	34	9	2	8	302	N/A	C3	Beijing, Chaoyang	2019-09-13	Water: River
GZBYW43.3	8	6	34	9	2	8	302	N/A	C3	Guangzhou, Yuexiu	2019-09-16	Water: Lake
GZBYW49.1	8	6	34	9	2	8	302	N/A	C3	Guangzhou, Yuexiu	2019-10-26	Water: Lake
GZYXW35.1	8	6	34	9	2	8	302	N/A	C3	Guangzhou, Yuexiu	2019-08-25	Water: Lake
GZYXW35.10	8	6	34	9	2	8	302	N/A	C3	Guangzhou, Yuexiu	2019-08-25	Water: Lake
GZYXW35.20	8	6	34	9	2	8	302	N/A	C3	Guangzhou, Yuexiu	2019-08-25	Water: Lake
GZYXW35.30	8	6	34	9	2	8	302	N/A	C3	Guangzhou, Yuexiu	2019-08-25	Water: Lake
GZYXW37.1	8	6	34	9	2	8	302	N/A	C3	Guangzhou, Yuexiu	2019-08-25	Water: Lake
GZYXW37.7	8	6	34	9	2	8	302	N/A	C3	Guangzhou, Yuexiu	2019-08-25	Water: Lake
MZDPW5.1	8	6	34	9	2	8	302	N/A	C3	Meizhou, Dapu	2019-10-02	Water: Small stream
MZDPW5.10	8	6	34	9	2	8	302	N/A	C3	Meizhou, Dapu	2019-10-02	Water: Small stream
MZDPW5.20	8	6	34	9	2	8	302	N/A	C3	Meizhou, Dapu	2019-10-02	Water: Small stream
MZDPW5.30	8	6	34	9	2	8	302	N/A	C3	Meizhou, Dapu	2019-10-02	Water: Small stream
MZDPW5.40	8	6	34	9	2	8	302	N/A	C3	Meizhou, Dapu	2019-10-02	Water: Small stream
MZDPW5.50	8	6	34	9	2	8	302	N/A	C3	Meizhou, Dapu	2019-10-02	Water: Small stream
QYYDW9.1	8	6	34	9	2	8	302	N/A	C3	Qingyuan, Yingde	2019-06-29	Water: Pond
SZGMW13.11	8	6	34	9	2	8	302	N/A	C3	Shenzhen, Guangming	2019-05-10	Water: Small stream
SZGMW28.9	8	6	34	9	2	8	302	N/A	C3	Shenzhen, Guangming	2019-05-17	Water: Pond
SZGMW63.17	8	6	34	9	2	8	302	N/A	C3	Shenzhen, Guangming	2019-07-19	Water: Lake
SZGMW66.9	8	6	34	9	2	8	302	N/A	C3	Shenzhen, Guangming	2019-07-28	Water: Lake
SZGMW74.10	8	6	34	9	2	8	302	N/A	C3	Shenzhen, Guangming	2019-07-28	Water: Lake
GZYXW39.1	2	3	5	10	2	1	302	N/A	C4	Guangzhou, Yuexiu	2019-08-25	Water: Lake
SZFTW77.19	2	3	5	10	2	1	302	N/A	C4	Shenzhen, Futian	2019-08-04	Water: Sea
SZFTW80.15	2	3	5	10	2	1	302	N/A	C4	Shenzhen, Futian	2019-08-04	Water: Sea
SZGMS1.10	2	3	5	10	2	1	302	N/A	C4	Shenzhen, Guangming	2019-12-12	Soil: Garden soil
GZZCS68.16	2	3	5	10	2	1	302	N/A	C4	Guangzhou, Zengcheng	2020-10-02	Soil: Garden soil
SZGMW57.1	6	35	14	13	2	201	304	N/A	C5	Shenzhen, Guangming	2019-07-19	Water: Lake
GZBYS23.1	38	57	35	13	2	202	305	N/A	C6	Guangzhou, Baiyun	2019-11-24	Soil: Garden soil
GZBYS23.10	38	57	35	13	2	202	305	N/A	C6	Guangzhou, Baiyun	2019-11-24	Soil: Garden soil
GZBYS23.7	38	57	35	13	2	202	305	N/A	C6	Guangzhou, Baiyun	2019-11-24	Soil: Garden soil
GZBYS23.9	38	57	35	13	2	202	305	N/A	C6	Guangzhou, Baiyun	2019-11-24	Soil: Garden soil
GZHZS8.2	8	201	34	47	2	8	302	N/A	C7	Guangzhou, Haizhu	2019-11-03	Soil: potted soil
GZHZS8.4	8	201	34	47	2	8	302	N/A	C7	Guangzhou, Haizhu	2019-11-03	Soil: potted soil
SZGMW111.2	8	201	34	47	2	8	302	N/A	C7	Shenzhen, Guangming	2020-09-22	Water: Small stream
GZYXS29.1	2	3	6	69	2	1	306	N/A	C8	Guangzhou, Yuexiu	2019-12-01	Soil: Potted soil
GZYXS37.1	2	3	6	69	2	1	306	N/A	C8	Guangzhou, Yuexiu	2019-12-01	Soil: Potted soil
SZGMS6.1	6	10	5	10	9	1	302	N/A	C9	Shenzhen, Guangming	2020-01-07	Soil: Garden soil
SZGMS1.6	6	10	14	11	9	14	302	N/A	C10	Shenzhen, Guangming	2019-12-12	Soil: Garden soil
GZBYS51.10	6	10	14	28	9	4	307	N/A	C11	Guangzhou, Baiyun	2019-12-08	Soil: Potted soil
SZGMS6.29	6	10	5	11	10	1	302	N/A	C12	Shenzhen, Guangming	2020-01-07	Soil: Garden soil
SZGMS6.33	6	10	5	11	10	1	302	N/A	C12	Shenzhen, Guangming	2020-01-07	Soil: Garden soil
HSXLW13.1	201	202	17	6	12	15	308	N/A	C13	Huangshi, Xialu	2019-06-13	Water: Lake
GZHZS9.1	7	10	17	23	13	11	301	N/A	C14	Guangzhou, Haizhu	2019-11-03	Soil: Potted soil
GZHZS9.10	7	10	17	23	13	11	301	N/A	C14	Guangzhou, Haizhu	2019-11-03	Soil: Potted soil
GZHZS9.13	7	10	17	23	13	11	301	N/A	C14	Guangzhou, Haizhu	2019-11-03	Soil: Potted soil
GZHZS9.15	7	10	17	23	13	11	301	N/A	C14	Guangzhou, Haizhu	2019-11-03	Soil: Potted soil
GZBYS51.6	6	10	14	28	21	14	309	N/A	C15	Guangzhou, Haizhu	2019-12-08	Soil: Potted soil
HSXLW15.10	6	10	15	28	21	7	310	N/A	C16	Huangshi, Xialu	2019-06-13	Water: Fountain
GZBYW10.1	8	6	34	9	53	8	302	N/A	C17	Guangzhou, Baiyun	2019-05-19	Water: Pond
GZBYW10.10	8	6	34	9	53	8	302	N/A	C17	Guangzhou, Baiyun	2019-05-19	Water: Pond
GZBYW49.20	8	6	34	9	53	8	302	N/A	C17	Guangzhou, Baiyun	2019-10-26	Water: Lake
GZBYW51.1	8	6	34	9	53	8	302	N/A	C17	Guangzhou, Baiyun	2019-10-26	Water: Lake
GZBYW51.10	8	6	34	9	53	8	302	N/A	C17	Guangzhou, Baiyun	2019-10-26	Water: Lake
GZBYW52.10	8	6	34	9	53	8	302	N/A	C17	Guangzhou, Baiyun	2019-10-26	Water: Lake
GZBYW52.16	8	6	34	9	53	8	302	N/A	C17	Guangzhou, Baiyun	2019-10-26	Water: Lake
GZBYW54.1	8	6	34	9	53	8	302	N/A	C17	Guangzhou, Baiyun	2019-10-26	Water: Lake
GZBYW54.10	8	6	34	9	53	8	302	N/A	C17	Guangzhou, Baiyun	2019-10-26	Water: Lake
GZBYW54.7	8	6	34	9	53	8	302	N/A	C17	Guangzhou, Baiyun	2019-10-26	Water: Lake
SZGMW96.2	8	6	34	9	53	8	302	N/A	C17	Shenzhen, Guangming	2020-03-02	Water: Small stream
GZBYW5.1	8	6	34	9	53	8	311	N/A	C18	Guangzhou, Baiyun	2019-05-12	Water: Pond
GZHZS9.3	202	203	9	52	67	202	312	N/A	C19	Guangzhou, Haizhu	2019-11-03	Soil: Potted soil
GZBYW44.2	12	6	9	52	67	203	305	N/A	C20	Guangzhou, Baiyun	2019-09-16	Water: Lake
BJCYW14.6	36	205	59	61	75	44	313	N/A	C21	Beijing, Chaoyang	2019-09-13	Water: River
SZGMW9.2	2	3	18	40	1	1	2	N/A	C22	Shenzhen, Guangming	2019-04-02	Water: Small stream
SZGMW50.1	6	35	14	13	2	201	2	N/A	C23	Shenzhen, Guangming	2019-07-09	Water: Pond
HSXSSS14.28	12	29	11	23	29	26	2	N/A	C24	Huangshi, Xisaishan	2020-04-09	Soil: Garden soil
NJPKW5.19	4	8	11	40	29	12	2	N/A	C25	Nanjing, Pukou	2019-07-28	Water: River
SZGMS6.14	17	23	13	23	32	22	2	N/A	C26	Shenzhen, Guangming	2020-01-07	Soil: Garden soil
SZGMS1.13	17	23	13	23	32	22	2	N/A	C26	Shenzhen, Guangming	2019-12-12	Soil: Garden soil
SZGMS6.11	17	23	13	23	32	22	2	N/A	C26	Shenzhen, Guangming	2020-01-07	Soil: Garden soil
SZGMS6.22	17	23	13	23	32	22	2	N/A	C26	Shenzhen, Guangming	2020-01-07	Soil: Garden soil
CDDJYW1.2	3	10	1	28	2	9	3	N/A	C27	Chengdu, Jingjiang	2019-10-30	Water: Lake
GZYXW35.25	6	8	49	12	4	4	3	N/A	C28	Guangzhou, Yuexiu	2019-08-25	Water: Lake
GZYXW35.37	6	8	49	12	4	4	3	N/A	C28	Guangzhou, Yuexiu	2019-08-25	Water: Lake
NJPKW2.1	6	8	49	12	4	4	3	N/A	C28	Nanjing, Pukou	2019-07-28	Water: River
SZGMW73.5	6	8	49	12	4	4	3	N/A	C28	Shenzhen, Guangming	2019-07-28	Water: River
GZBYS47.21	203	206	202	13	202	5	6	N/A	C29	Guangzhou, Baiyun	2019-12-08	Soil: Garden soil
SZGMW66.23	27	3	9	201	2	1	6	N/A	C30	Shenzhen, Guangming	2019-07-28	Water: Lake
SZGMW28.7	8	6	34	9	2	8	6	N/A	C31	Shenzhen, Guangming	2019-05-17	Water: Pond
SZGMW68.1	8	6	34	9	2	8	6	N/A	C31	Shenzhen, Guangming	2019-07-28	Water: Lake
SZGMW7.1	8	6	34	9	2	8	6	N/A	C31	Shenzhen, Guangming	201/4/26	Water: Small stream
SZGMW73.1	8	6	34	9	2	8	6	N/A	C31	Shenzhen, Guangming	2019-07-28	Water: River
SZGMW2.1	1	3	9	10	2	6	6	N/A	C32	Shenzhen, Guangming	2019-04-26	Water: Small stream
SJJZDS4.12	2	3	5	10	2	6	6	N/A	C33	Shijiazhuang, Zhengding	2019-10-17	Soil: Garden soil
SJJZDS4.9	2	3	5	10	2	6	6	N/A	C33	Shijiazhuang, Zhengding	2019-10-17	Soil: Garden soil
SJZZDS4.10	2	3	5	10	2	6	6	N/A	C33	Shijiazhuang, Zhengding	2019-10-17	Soil: Garden soil
SJZZDS4.7	2	3	5	10	2	6	6	N/A	C33	Shijiazhuang, Zhengding	2019-10-17	Soil: Garden soil
SZGMS4.39	2	3	5	12	2	6	6	N/A	C34	Shenzhen, Guangming	2020-01-07	Soil: Garden soil

SZGMW74.1	2	3	6	12	2	1	6	N/A	C35	Shenzhen, Guangming	2019-07-28	Water: Lake
GZBYS47.106	2	3	9	13	2	5	6	N/A	C36	Guangzhou, Baiyun	2019-12-08	Soil: Garden soil
GZBYS47.5	2	3	9	13	2	5	6	N/A	C36	Guangzhou, Baiyun	2019-12-08	Soil: Garden soil
GZBYS47.53	2	3	9	13	2	5	6	N/A	C36	Guangzhou, Baiyun	2019-12-08	Soil: Garden soil
BJCYW12.5	2	10	57	13	2	6	6	N/A	C37	Beijing, Chaoyang	2019-09-13	Water: River
GZBYS47.10	2	207	40	69	2	5	6	N/A	C38	Guangzhou, Baiyun	2019-12-08	Soil: Garden soil
GZBYS47.107	2	207	40	69	2	5	6	N/A	C38	Guangzhou, Baiyun	2019-12-08	Soil: Garden soil
GZBYS47.18	2	207	40	69	2	5	6	N/A	C38	Guangzhou, Baiyun	2019-12-08	Soil: Garden soil
GZBYS47.19	2	207	40	69	2	5	6	N/A	C38	Guangzhou, Baiyun	2019-12-08	Soil: Garden soil
GZBYS47.27	2	207	40	69	2	5	6	N/A	C38	Guangzhou, Baiyun	2019-12-08	Soil: Garden soil
GZBYS47.29	2	207	40	69	2	5	6	N/A	C38	Guangzhou, Baiyun	2019-12-08	Soil: Garden soil
GZBYS47.33	2	207	40	69	2	5	6	N/A	C38	Guangzhou, Baiyun	2019-12-08	Soil: Garden soil
GZBYS47.4	2	207	40	69	2	5	6	N/A	C38	Guangzhou, Baiyun	2019-12-08	Soil: Garden soil
GZBYS47.44	2	207	40	69	2	5	6	N/A	C38	Guangzhou, Baiyun	2019-12-08	Soil: Garden soil
GZBYS47.49	2	207	40	69	2	5	6	N/A	C38	Guangzhou, Baiyun	2019-12-08	Soil: Garden soil
GZBYS47.65	2	207	40	69	2	5	6	N/A	C38	Guangzhou, Baiyun	2019-12-08	Soil: Garden soil
GZBYS47.68	2	207	40	69	2	5	6	N/A	C38	Guangzhou, Baiyun	2019-12-08	Soil: Garden soil
GZBYS47.73	2	207	40	69	2	5	6	N/A	C38	Guangzhou, Baiyun	2019-12-08	Soil: Garden soil
GZBYS47.77	2	207	40	69	2	5	6	N/A	C38	Guangzhou, Baiyun	2019-12-08	Soil: Garden soil
GZBYS47.84	2	207	40	69	2	5	6	N/A	C38	Guangzhou, Baiyun	2019-12-08	Soil: Garden soil
GZBYS47.95	2	207	40	69	2	5	6	N/A	C38	Guangzhou, Baiyun	2019-12-08	Soil: Garden soil
GZBYS47.98	2	207	40	69	2	5	6	N/A	C38	Guangzhou, Baiyun	2019-12-08	Soil: Garden soil
GZBYS17.2	2	10	14	3	18	4	6	N/A	C39	Guangzhou, Baiyun	2019-11-17	Soil: Garden soil
SZGMS3.1	6	10	14	12	18	14	6	N/A	C40	Shenzhen, Guangming	2020-01-07	Soil: Garden soil
SZGMS6.32	2	10	5	28	18	14	6	N/A	C41	Shenzhen, Guangming	2020-01-07	Soil: Garden soil
SZGMS6.7	2	10	5	28	18	14	6	N/A	C41	Shenzhen, Guangming	2020-01-07	Soil: Garden soil
SZGMS4.25	6	10	5	28	18	4	6	N/A	C42	Shenzhen, Guangming	2020-01-07	Soil: Garden soil
HSXSSS14.2	2	10	5	47	18	5	6	N/A	C43	Huangshi, Xisaishan	2020-04-09	Soil: Garden soil
HSXSSS14.14	2	10	5	47	18	5	6	N/A	C43	Huangshi, Xisaishan	2020-04-09	Soil: Garden soil
HSXSSS14.20	2	10	5	47	18	5	6	N/A	C43	Huangshi, Xisaishan	2020-04-09	Soil: Garden soil
HSXSSS14.3	2	10	5	47	18	5	6	N/A	C43	Huangshi, Xisaishan	2020-04-09	Soil: Garden soil
HSXSSS14.33	2	10	5	47	18	5	6	N/A	C43	Huangshi, Xisaishan	2020-04-09	Soil: Garden soil
SZGMS6.13	2	10	5	48	18	14	6	N/A	C44	Shenzhen, Guangming	2020-01-07	Soil: Garden soil
GZYXW39.55	2	3	6	13	56	1	6	N/A	C45	Guangzhou, Yuexiu	2019-08-25	Water: Lake
SZFTW83.1	2	3	9	13	63	1	6	N/A	C46	Shenzhen, Futian	2019-08-04	Water: Lake
SZGMW72.3	2	3	9	13	63	1	6	N/A	C46	Shenzhen, Guangming	2019-07-28	Water: Lake
SZGMW103.2	2	3	9	13	63	1	6	N/A	C46	Shenzhen, Guangming	2020-09-15	Water: Small stream
SZGMW104.1	2	3	9	13	63	1	6	N/A	C46	Shenzhen, Guangming	2020-09-15	Water: Small stream
SZGMW106.5	2	3	9	13	63	1	6	N/A	C46	Shenzhen, Guangming	2020-09-22	Water: Small stream
GZHZS9.4	26	57	35	10	67	202	6	N/A	C47	Guangzhou, Haizhu	2019-11-03	Soil: Potted soil
GZBYW43.2	26	6	9	52	67	203	6	N/A	C48	Guangzhou, Baiyun	2019-09-16	Water: Lake
GZBYW48.1	26	6	9	52	67	203	6	N/A	C48	Guangzhou, Baiyun	2019-09-16	Water: Grassland puddle
SZGMW89.1	1	4	3	202	2	1	9	N/A	C49	Shenzhen, Guangming	2020-03-02	Water: Small river
GZBYS53.2	6	10	14	3	2	22	9	N/A	C50	Guangzhou, Baiyun	2019-12-08	Soil: Potted soil
SZGMW63.28	8	6	34	9	2	8	303	N/A	C51	Shenzhen, Guangming	2019-07-19	Water: Lake
SZGMW65.2	3	10	1	1	14	9	9	N/A	C52	Shenzhen, Guangming	2019-07-18	Water: Lake
SZGMW70.1	3	10	1	1	14	9	9	N/A	C52	Shenzhen, Guangming	2019-07-18	Water: Lake
SZGMS2.16	2	10	23	203	18	14	9	N/A	C53	Shenzhen, Guangming	2019-12-12	Soil: Garden soil
GZBYS61.2	2	10	5	3	18	14	9	N/A	C54	Guangzhou, Baiyun	2019-12-27	Soil: Garden soil
SZGMS2.10	2	10	5	3	18	14	9	N/A	C54	Shenzhen, Guangming	2019-12-12	Soil: Garden soil
SZGMS2.13	2	10	5	3	18	14	9	N/A	C54	Shenzhen, Guangming	2019-12-12	Soil: Garden soil
SZGMS2.15	2	10	5	3	18	14	9	N/A	C54	Shenzhen, Guangming	2019-12-12	Soil: Garden soil
SZGMS2.18	2	10	5	3	18	14	9	N/A	C54	Shenzhen, Guangming	2019-12-12	Soil: Garden soil
SZGMS4.11	2	10	5	3	18	14	9	N/A	C54	Shenzhen, Guangming	2020-01-07	Soil: Garden soil
SZGMS4.18	2	10	5	3	18	14	9	N/A	C54	Shenzhen, Guangming	2020-01-07	Soil: Garden soil
SZGMS4.2	2	10	5	3	18	14	9	N/A	C54	Shenzhen, Guangming	2020-01-07	Soil: Garden soil
SZGMS4.20	2	10	5	3	18	14	9	N/A	C54	Shenzhen, Guangming	2020-01-07	Soil: Garden soil
SZGMS4.43	2	10	5	3	18	14	9	N/A	C54	Shenzhen, Guangming	2020-01-07	Soil: Garden soil
GZBYS64.1	2	10	5	10	18	14	9	N/A	C55	Guangzhou, Baiyun	2019-12-27	Soil: Garden soil
SZGMS4.36	2	10	5	10	18	14	9	N/A	C55	Shenzhen, Guangming	2020-01-07	Soil: Garden soil
GZBYS17.3	6	10	14	40	21	14	9	N/A	C56	Shenzhen, Guangming	2019-11-17	Soil: Garden soil
SZGMS2.4	17	23	13	48	32	22	9	N/A	C57	Shenzhen, Guangming	2019-12-12	Soil: Garden soil
SZGMW39.1	12	8	203	204	40	1	9	N/A	C58	Shenzhen, Guangming	2019-07-04	Water: Grassland puddle
SZGMS1.9	5	1	22	74	50	10	9	N/A	C59	Shenzhen, Guangming	2019-12-12	Soil: Garden soil
SZGMW73.18	8	6	34	9	2	8	11	N/A	C60	Shenzhen, Guangming	2019-07-28	Water: River
SZGMW34.5	6	6	48	3	9	14	11	N/A	C61	Shenzhen, Guangming	2019-07-04	Water: Pond
HSXLW20.1	32	6	48	6	12	50	11	N/A	C62	Shenzhen, Guangming	2019-06-13	Water: Fountain
QYYDW7.1	32	6	48	6	12	50	11	N/A	C62	Qingyuan, Yingde	2019-06-29	Water: Pond
QYYDW7.5	32	6	48	6	12	50	11	N/A	C62	Qingyuan, Yingde	2019-06-29	Water: Pond
HSXLW16.1	32	6	48	6	21	50	11	N/A	C63	Huangshi, Xialu	2019-06-13	Water: Fountain
SZFTW79.1	32	208	204	205	48	204	11	N/A	C64	Shenzhen, Futian	2019-08-04	Water: Sea
SZGMW65.11	2	10	9	10	2	1	12	N/A	C65	Shenzhen, Guangming	2019-07-28	Water: Lake
GZBYS47.12	5	1	22	30	6	10	12	N/A	C66	Guangzhou, Baiyun	2019-12-08	Soil: Garden soil
GZBYS47.41	5	1	22	30	6	10	12	N/A	C66	Guangzhou, Baiyun	2019-12-08	Soil: Garden soil
SZGMS2.2	17	23	13	10	91	22	12	N/A	C67	Shenzhen, Guangming	2019-12-12	Soil: Garden soil
SZGMS4.32	26	9	2	5	207	17	15	N/A	C68	Shenzhen, Guangming	2020-01-07	Soil: Garden soil
BJCYW11.7	2	44	5	5	203	1	15	N/A	C69	Beijing, Chaoyang	2019-09-13	Water: River
BJCYW12.4	2	44	5	5	203	1	15	N/A	C69	Beijing, Chaoyang	2019-09-13	Water: River
BJCYW11.2	8	6	34	9	2	8	15	N/A	C70	Beijing, Chaoyang	2019-09-13	Water: River
SZGMS6.20	12	29	2	12	3	17	15	N/A	C71	Shenzhen, Guangming	2020-01-07	Soil: Garden soil
DLSHKW9.1	3	10	1	28	14	9	15	N/A	C72	Dalian, Shihekou	2019-08-23	Water: River
SZGMS5.1	2	10	14	206	18	205	15	N/A	C73	Shenzhen, Guangming	2020-01-07	Soil: Garden soil
SZGMS4.28	2	10	5	10	18	14	15	N/A	C74	Shenzhen, Guangming	2020-01-07	Soil: Garden soil
SZGMS4.27	12	209	205	48	18	17	15	N/A	C75	Shenzhen, Guangming	2020-01-07	Soil: Garden soil
GZBYW40.1	202	203	201	13	67	203	305	N/A	C76	Guangzhou, Yuexiu	2019-09-16	Water: Lake
GZBYW43.1	202	203	201	13	67	203	305	N/A	C76	Guangzhou, Yuexiu	2019-09-16	Water: Lake
GZBYW48.2	202	203	201	13	67	203	305	N/A	C76	Guangzhou, Yuexiu	2019-09-16	Water: Grassland puddle
HSXSSS14.10	12	8	2	5	50	20	15	N/A	C77	Huangshi, Xisaishan	2020-04-09	Soil: Garden soil
HZHYW4.2	12	29	26	5	50	20	15	N/A	C78	Huizhou, Huiyang	2019-10-31	Water: Lake
GZBYW44.1	4	15	11	16	30	206	24	N/A	C79	Guangzhou, Baiyun	2019-09-16	Water: Lake
SZFTW77.3	4	8	11	5	29	12	34	N/A	C80	Shenzhen, Futian	2019-08-04	Water: Sea
SZGMW19.1	5	1	22	30	6	10	37	N/A	C81	Shenzhen, Guangming	2019-05-10	Water: Small stream
SZGMW11.1	8	6	34	9	18	8	37	N/A	C82	Shenzhen, Guangming	2019-05-10	Water: Small stream
SZGMS4.1	4	15	11	23	30	12	37	N/A	C83	Shenzhen, Guangming	2020-01-07	Soil: Garden soil
GZBYW2.1	8	6	34	9	53	8	37	N/A	C84	Guangzhou, Baiyun	2019-05-12	Water: Pond
GZYXW38.1	2	201	31	6	12	15	38	N/A	C85	Guangzhou, Yuexiu	2019-08-25	Water: Lake
GZYXW38.10	2	201	31	6	12	15	38	N/A	C85	Guangzhou, Yuexiu	2019-08-25	Water: Lake
GZYXW38.20	2	201	31	6	12	15	38	N/A	C85	Guangzhou, Yuexiu	2019-08-25	Water: Lake

GZYXW38.30	2	201	31	6	12	15	38	N/A	C85	Guangzhou, Yuexiu	2019-08-25	Water: Lake
GZYXW38.50	2	201	31	6	12	15	38	N/A	C85	Guangzhou, Yuexiu	2019-08-25	Water: Lake
GZYXW38.60	2	201	31	6	12	15	38	N/A	C85	Guangzhou, Yuexiu	2019-08-25	Water: Lake
GZYXW38.70	2	201	31	6	12	15	38	N/A	C85	Guangzhou, Yuexiu	2019-08-25	Water: Lake
SZFTW84.1	2	201	31	6	12	15	38	N/A	C85	Shenzhen, Futian	2019-08-04	Water: Lake
SZFTW84.10	2	201	31	6	12	15	38	N/A	C85	Shenzhen, Futian	2019-08-04	Water: Lake
SZFTW84.16	2	201	31	6	12	15	38	N/A	C85	Shenzhen, Futian	2019-08-04	Water: Lake
SZFTW84.3	2	201	31	6	12	15	38	N/A	C85	Shenzhen, Futian	2019-08-04	Water: Lake
SZFTW84.7	2	201	31	6	12	15	38	N/A	C85	Shenzhen, Futian	2019-08-04	Water: Lake
SZFTW85.1	2	201	31	6	12	15	38	N/A	C85	Shenzhen, Futian	2019-08-04	Water: Lake
SZFTW85.14	2	201	31	6	12	15	38	N/A	C85	Shenzhen, Futian	2019-08-04	Water: Lake
SZGMW34.1	2	201	31	6	12	15	38	N/A	C85	Shenzhen, Guangming	2019-07-04	Water: Pond
SZGMW105.4	2	**201**	31	6	12	15	38	N/A	C85	Shenzhen, Guangming	2020-09-15	Water: Small stream
QYYDW7.3	32	6	48	6	48	5	38	N/A	C86	Qingyuan, Yingde	2019-06-29	Water: Pond
SZGMW12.1	2	10	14	10	18	4	40	N/A	C87	Shenzhen, Guangming	2019-05-10	Water: Small stream
QYYDW8.1	32	53	17	6	12	15	54	N/A	C88	Qingyuan, Yingde	2019-06-29	Water: Pond
SZGMS1.1	17	23	13	48	32	22	205	N/A	C89	Shenzhen, Guangming	2019-12-12	Soil: Garden soil
SZGMS1.12	17	23	13	48	32	22	205	N/A	C89	Shenzhen, Guangming	2019-12-12	Soil: Garden soil
SZGMS1.5	17	23	13	48	32	22	205	N/A	C89	Shenzhen, Guangming	2019-12-12	Soil: Garden soil
SZGMS1.7	17	23	13	48	32	22	205	N/A	C89	Shenzhen, Guangming	2019-12-12	Soil: Garden soil
SZGMS4.12	17	23	13	48	32	22	205	N/A	C89	Shenzhen, Guangming	2020-01-07	Soil: Garden soil
SZGMS4.19	17	23	13	48	32	22	205	N/A	C89	Shenzhen, Guangming	2020-01-07	Soil: Garden soil
SZGMS5.3	17	23	13	48	32	22	205	N/A	C89	Shenzhen, Guangming	2020-01-07	Soil: Garden soil
SZGMS6.24	17	23	13	48	32	22	205	N/A	C89	Shenzhen, Guangming	2020-01-07	Soil: Garden soil
SZGMS7.1	17	23	13	48	32	22	205	N/A	C89	Shenzhen, Guangming	2020-01-07	Soil: Garden soil
SZGMS2.8	17	23	13	48	91	22	205	N/A	C90	Shenzhen, Guangming	2019-12-12	Soil: Garden soil
BJCYW11.8	30	41	55	51	15	44	206	N/A	C91	Beijing, Chaoyang	2019-09-13	Water: River
GZBYS61.1	2	10	23	206	18	14	207	N/A	C92	Shenzhen, Guangming	2019-12-27	Soil: Garden soil
SZGMS3.3	2	10	2	10	18	14	207	N/A	C93	Shenzhen, Guangming	2020-01-07	Soil: Garden soil
SZGMS9.4	2	10	14	10	18	14	207	N/A	C94	Shenzhen, Guangming	2020-01-07	Soil: Garden soil
SZGMS1.2	6	10	5	10	18	14	207	N/A	C95	Shenzhen, Guangming	2019-12-12	Soil: Garden soil
BJCYW15.2	6	10	3	13	19	4	207	N/A	C96	Beijing, Chaoyang	2019-09-13	Water: River
BJCYW15.1	6	10	15	13	19	4	207	N/A	C97	Beijing, Chaoyang	2019-09-13	Water: River
SZGMS1.14	2	10	5	3	4	14	213	N/A	C98	Shenzhen, Guangming	2019-12-12	Soil: Garden soil
BJCYW13.1	10	8	7	5	16	18	213	N/A	C99	Beijing, Chaoyang	2019-09-13	Water: River
BJCYW13.13	10	8	7	5	16	18	213	N/A	C99	Beijing, Chaoyang	2019-09-13	Water: River
BJCYW13.17	10	8	7	5	16	18	213	N/A	C99	Beijing, Chaoyang	2019-09-13	Water: River
BJCYW13.6	10	8	7	5	16	18	213	N/A	C99	Beijing, Chaoyang	2019-09-13	Water: River
BJCYW13.8	10	8	7	5	16	18	213	N/A	C99	Beijing, Chaoyang	2019-09-13	Water: River
BJCYW14.1	10	8	7	13	16	18	213	N/A	C100	Beijing, Chaoyang	2019-09-13	Water: River
HSXSSS14.22	12	31	26	5	50	20	213	N/A	C101	Huangshi, Xisaishan	2020-04-09	Soil: Garden soil
GZBYW51.19	29	49	30	9	48	31	214	N/A	C102	Guangzhou, Baiyun	2019-10-26	Water: Lake
GZBYW52.1	29	49	30	9	48	31	214	N/A	C102	Guangzhou, Baiyun	2019-10-26	Water: Lake
HSXLW13.20	29	49	30	9	48	31	214	N/A	C102	Huangshi, Xialu	2019-06-13	Water: Lake
SZFTW76.17	29	49	30	9	48	31	214	N/A	C102	Shenzhen, Futian	2019-08-04	Water: Sea
DGSSHW4.1	12	57	9	207	67	14	218	N/A	C103	Dongguan, Songshanhu	2019-08-18	Water: Lake
SZFTW76.10	3	12	1	6	1	9	220	N/A	C104	Shenzhen, Futian	2019-08-04	Water: Sea
SZGMW70.2	2	12	22	6	48	11	220	N/A	C105	Shenzhen, Guangming	2019-07-28	Water: Lake
SZGMW36.1	32	3	17	6	12	31	229	N/A	C106	Shenzhen, Guangming	2019-07-04	Water: Pond
GZBYW49.10	8	6	17	6	12	15	229	N/A	C107	Guangzhou, Baiyun	2019-10-26	Water: Lake
HSXLW12.1	8	6	17	6	12	15	229	N/A	C107	Huangshi, Xialu	2019-06-13	Water: Lake
HSXLW13.10	8	6	17	6	12	15	229	N/A	C107	Huangshi, Xialu	2019-06-13	Water: Lake
HSXLW13.29	8	6	17	6	12	15	229	N/A	C107	Huangshi, Xialu	2019-06-13	Water: Lake
HSXSSW21.28	8	6	17	6	12	15	229	N/A	C107	Huangshi, Xisaishan	2019-06-14	Water: River
HSXSSW21.27	8	6	17	6	12	50	229	N/A	C108	Huangshi, Xisaishan	2019-06-14	Water: River
SZGMW99.1	3	6	3	3	9	4	207	N/A	C109	Shenzhen, Guangming	2020-09-15	Water: Small stream
SZGMW99.3	38	57	9	10	208	202	40	N/A	C110	Shenzhen, Guangming	2020-09-15	Water: Small stream
SZGMW101.1	7	6	17	3	209	11	40	N/A	C111	Shenzhen, Guangming	2020-09-15	Water: Small stream
SZGMW103.1	2	3	3	208	2	20	218	N/A	C112	Shenzhen, Guangming	2020-09-15	Water: Small stream
SZGMW103.10	12	8	11	209	40	12	2	N/A	C113	Shenzhen, Guangming	2020-09-15	Water: Small stream
SZGMW103.15	2	3	9	13	204	5	11	N/A	C114	Shenzhen, Guangming	2020-09-15	Water: Small stream
SZGMW103.17	2	210	9	210	205	5	218	N/A	C115	Shenzhen, Guangming	2020-09-15	Water: Small stream
SZGMW103.25	5	1	22	74	20	10	203	N/A	C115	Shenzhen, Guangming	2020-09-15	Water: Small stream
SZGMW107.1	3	13	1	28	14	9	11	N/A	C117	Shenzhen, Guangming	2020-09-22	Water: Small stream
SZGMW108.3	3	13	1	28	14	9	11	N/A	C117	Shenzhen, Guangming	2020-09-22	Water: Small stream
SZGMW110.2	3	13	1	28	14	9	11	N/A	C117	Shenzhen, Guangming	2020-09-22	Water: Small stream
SZGMW107.3	3	13	1	3	14	9	9	N/A	C118	Shenzhen, Guangming	2020-09-22	Water: Small stream
SZGMW111.1	12	6	9	52	67	207	305	N/A	C119	Shenzhen, Guangming	2020-09-22	Water: Small stream
SZGMW115.1	6	10	19	28	21	14	9	N/A	C120	Shenzhen, Guangming	2020-10-07	Water: Pond
SZGMS16.1	2	3	6	11	206	1	6	N/A	C121	Shenzhen, Guangming	2020-05-08	Soil: Garden soil
SZGMS16.10	2	3	6	11	206	1	6	N/A	C121	Shenzhen, Guangming	2020-05-08	Soil: Garden soil
SZGMS16.20	2	3	6	11	206	1	6	N/A	C121	Shenzhen, Guangming	2020-05-08	Soil: Garden soil
SZGMS16.30	2	3	6	11	206	1	6	N/A	C121	Shenzhen, Guangming	2020-05-08	Soil: Garden soil
SZGMS16.40	2	3	6	11	206	1	6	N/A	C121	Shenzhen, Guangming	2020-05-08	Soil: Garden soil
GZZCS68.1	4	8	11	10	40	12	6	N/A	C122	Guangzhou, Zengcheng	2020-10-02	Soil: Garden soil
GZZCS68.10	4	8	11	10	40	12	6	N/A	C122	Guangzhou, Zengcheng	2020-10-02	Soil: Garden soil
BJCYW13.10	2	3	9	10	2	8	6	N/A	C123	Beijing, Chaoyang	2019-09-13	Water: River
SZGMS5.5	2	3	9	10	2	5	218	N/A	C124	Shenzhen, Guangming	2020-01-07	Soil: Garden soil
SZGMW104.7	2	6	48	6	48	5	40	N/A	C125	Shenzhen, Guangming	2020-09-15	Water: Small stream
SZGMW106.1	6	10	15	28	21	14	207	N/A	C126	Shenzhen, Guangming	2020-09-22	Water: Small stream
SZGMW98.3	6	10	49	10	4	4	3	N/A	C127	Shenzhen, Guangming	2020-09-15	Water: Small stream
SJZZDS4.13	2	3	40	10	2	1	9	N/A	C128	Shijiazhuang, Zhengding	2019-10-17	Soil: Garden soil

and numbers marked red indicates novel allelic profiles found in this study and are temporary named or numbered due to the unavailability of the SBT server.

Table S4. Clonal complexes of STs derived from environmental sources of China.

Sources	Clonal complexes
All sources	C3, C20, C23, C62, C66, C89, C97, C99, C107, ST506#, ST260, ST461&, ST579, ST710, ST763
Water sources	C3, C20, C23, C62, C97, C99, C107, ST367&, ST506, ST579, ST763, ST1324*
Soil sources	C33#, C66, C89, ST710, ST260, ST461

* indicates that ST1324 is the founder of a complex in water STs when soil ST C66 was removed.

& indicates that ST367 is the founder ST of a complex in water STs, while this complex is part of ST461 complex in all STs.

# indicates that in soil STs, C33 complex is part of ST506 complex when water STs is removed.

Table S5. Summary of genetic diversity for each locusof *L. pneumophila* isolates from water& soil sources based on the concatenated SBT sequences.

	Water isolates							Soil isolates						
Parameters&Locus#	*flaA*	*pilE*	*asd*	*mip*	*momps*	*proA*	*neuA*	*flaA*	*pilE*	*asd*	*mip*	*momps*	*proA*	*neuA*
Sequences, n														
Haplotypes, h	19	23	26	31	28	24	32	13	17	17	23	23	17	21
Haplotype diversity, Hd	0.869	0.871	0.903	0.917	0.877	0.903	0.927	0.796	0.864	0.0900	0.929	0.878	0.900	0.864
Nucleotide diversity, π	0.0173&	0.0261	0.0101	0.0102	0.02305	0.01832	0.1807	**0.0242^**	**0.0354**	**0.0173**	0.0097	0.02704	0.0186	0.1548
(standard deviation)	0.0011	0.0010	0.0014	0.00037	0.00156	0.00035	0.00874	**0.0023**	**0.0017**	0.0014	0.00034	0.00194	0.0006	0.01168
Polymorphic sites, S	30	55	42	42	78	35	176	22	43	35	22	49	30	176
Theta per site (from S)	0.0271	0.0269	0.01451	0.01712	0.03646	0.01406	0.08157	0.0207	0.0218	0.1257	0.00933	0.02382	0.0125	0.08481
(standard deviation)	0.0074	0.0065	0.0037	0.0044	0.0085	0.0037	0.01777	0.0061	0.0057	0.0034	0.0028	0.0060	0.00347	0.01901
Average number of nucleotide differences, k	3.108	8.701	7.740	4.085	8.021	7.419	63.422	4.358	**11.796**	8.167	3.872	9.410	7.520	54.347
Total number of mutations, Eta	31	62	43	45	86	39	238	32	45	35	24	54	32	232
dN	0.00263	0.0060	0.00132	0.002403	0.01106	**0.01165**	0.1523	0.00267	0.00828	0.00291	0.001765	0.01075	**0.000755**	0.1352
dS	0.06217	0.09551	0.03947	0.04072	0.07573	0.08636	0.8080	0.09588	0.1342	0.06955	0.04050	0.1018	0.08952	0.6876
dN/dS	0.0423	0.0628	0.0334	0.05901	0.1460	**0.1349**	0.1885	0.0278	0.0617	0.0418	0.04356	0.1056	**0.0084**	0.1966
Tajima's D	-1.0627	-0.405	-0.9243	-1.273	-1.28411	0.4811	1.98655	0.04629	1.6151	1.0887	-0.1267	0.09035	1.1058	1.21815
Fu and Li's D*	1.2333	-2.198	-1.6052	-4.888**	2.1289**	1.5071	1.6247**	1.3241	1.9701**	2.1076**	-0.8777	1.3413	1.2961	1.8017**
Fu and Li's F*	0.31357	-1.652	-1.5723	-3.957**	0.6146	1.2775	2.1469**	1.1782	2.1846**	2.0179**	-0.6905	0.9453	1.4645	1.8068**

* P<0.05, **P<0.02

#To fit the codon frame for dN&dS analysis, 2-4 bp of nucleotide were removed from the locus *flaA* (2 bp),*asd* (2 bp), *mip* (3 bp) and *momps* (4bp).

& The blue color indicates that the values of the selected parameters are lower than the comparable ones in the isolates from another environmental sources.

^ The higher color indicates that the values of the selected parameters are higher than the comparable ones in the isolates from another environmental sources.

Table S6. Summary of AMOVA* results.

Source of variation	Sum of squares	Variance components	Percentage variation	Fixation index	Value	*P-value*
Among groups	183.486	-1.36809 (Va)	-2.79	FCT	-0.02791	0.66178
Among populations within groups	816.358	4.14126 (Vb)	8.45	FSC	0.08218	<0.0001
Within groups	16697.233	46.25272 (Vc)	94.34	FST	0.05657	<0.0001
Total	17697.077	49.02589	100	N/A	N/A	N/A

*AMOVA testing included two groups consisting of isolates from Guangzhou and Shenzhen cities and these isolates were split into two populations (water and soil isolates).

N/A: not available
